# Near-Infrared Spectroscopy and Machine Learning for Accurate Dating of Historical Books

**DOI:** 10.1021/jacs.3c02835

**Published:** 2023-05-22

**Authors:** Floriana Coppola, Luca Frigau, Jernej Markelj, Jasna Malešič, Claudio Conversano, Matija Strlič

**Affiliations:** † Faculty of Chemistry and Chemical Technology, University of Ljubljana, Večna pot 113, Ljubljana 1000, Slovenia; ‡ Department of Business and Economics, University of Cagliari, Via Sant’Ignazio da Laconi 17, Cagliari 09123, Italy; § National and University Library of Slovenia, Turjaška ulica 1, Ljubljana 1000, Slovenia; ∥ Institute for Sustainable Heritage, University College London, 14 Upper Woburn Place, London WC1H 0NN, U.K.

## Abstract

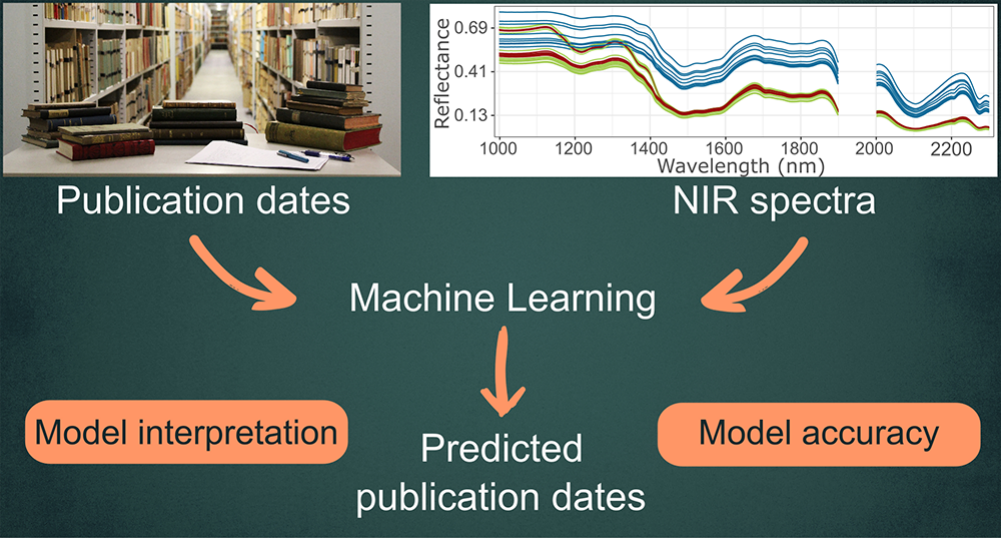

Non-destructive, fast, and accurate methods of dating are highly desirable for many heritage objects. Here, we present and critically evaluate the use of near-infrared (NIR) spectroscopic data combined with three supervised machine learning methods to predict the publication year of paper books dated between 1851 and 2000. These methods provide different accuracies; however, we demonstrate that the underlying processes refer to common spectral features. Regardless of the machine learning method used, the most informative wavelength ranges can be associated with C–H and O–H stretching first overtone, typical of the cellulose structure, and N–H stretching first overtone from amide/protein structures. We find that the expected influence of degradation on the accuracy of prediction is not meaningful. The variance-bias decomposition of the reducible error reveals some differences among the three machine learning methods. Our results show that two out of the three methods allow predictions of publication dates in the period 1851–2000 from NIR spectroscopic data with an unprecedented accuracy of up to 2 years, better than any other non-destructive method applied to a real heritage collection.

## Introduction

Knowing the provenance or the time of creation of heritage objects is of significant importance for curatorial, conservation, and scientific reasons. Tens of thousands of undated paper documents, manuscripts, and books are preserved in libraries and archives.^1^ Dating of objects, especially those with great historical value, is often a disputed matter, as demonstrated by the still-debated dating of the infamous Vinland map,^2^ or by the persistent market of forgeries, such as the Hitler Diaries.^3^ Not surprisingly, from 2005 to 2006, the National Archives (London, UK) undertook an investigation in response to allegations that certain documents were forgeries, and 29 turned out to be forged documents.^4^

Several aspects of a document, including the style, provenance, references to eminent individuals or events, handwriting, type of carrier, watermarks, pigments, dyes and inks used, or sometimes even damages and mends, may assist with dating at least within a limited date range.^5,6^ A recent approach based on the analysis of watermarks reported very encouraging results for dating incunabula printed in Low Countries within a margin of error of 1 year.^1^ However, despite the exceptional accuracy, several fundamental hurdles must be overcome, such as the reconstitution of watermarks occurring in the inner margins of tightly sewn manuscripts, especially in the case of quarto and octavo formats; the time needed for categorizing the watermarks by similarity; and, first and foremost, their presence, mostly limited to European manually produced paper up to 19th century. Watermarking was introduced in Italy in the 13th century and soon became the general trade-marks of the papermakers to assure the quality and authenticity of paper. Watermarked paper is still in use today, particularly for high-quality and various specialty papers, such as banknotes. Analysis of watermarks and irregularities in chain and laid lines in paper can suggest an estimate of chronology and provenance for historical documents and works of art, such as etchings by Raphael^7^ and Rembrandt.^8^ However, as Hunter warns,^9^ a dated mold might have been used for many years with the same time interval, the papermaker not going through the trouble of changing it, thus resulting in a discrepancy of almost 50 years between the date of paper production and the time to which it was dated, respectively, 1810 and 1859.

Numerous other techniques, including radiocarbon measurements,^10^ have found application also in forensics^11^ for dating paper documents, with some limitations for the more recent wood-derived papers. Using the so-called ″bomb peak″, a sharp increase in radiocarbon concentration induced by nuclear detonation tests carried out after World War II, accuracy of up to 1 year can be reached. However, for wood-derived paper the age of the trees used to produce the paper should be known, information that is very hard to obtain.^10^ Moreover, ideally, methods to establish the provenance or to date heritage and art objects should be based on non-destructive techniques, with a high degree of accuracy.^12^ In general, methods based on vibrational spectroscopy have been extensively used for the characterization of heritage materials. In the last two decades, attempts have been made also at dating, in particular using near-infrared (NIR) spectroscopy, most commonly employed in diffuse reflectance mode. NIR spectroscopy offers the advantage of working with fiber-optic probes in close contact with the sample without having to apply pressure and provides information on both surface and bulk properties. When investigating how spectral features in the NIR region (780–2500 nm) reflect specific material chemical and physical attributes, one is confronted with highly convoluted spectra dominated by overtones and combination vibrations, especially of OH, CH, and NH functionalities. Cellulose, a linear homopolysaccharide, composed of β-D-glucopyranoside units linked by (1–4) bonds, with a very complex morphological structure (developing micro- and macrofibrils by hydrogen bonds), is the main structural component of paper,^13^ and can derive from different origins (e.g., cotton, groundwood, or bleached fibers). Other components, including lignin, sizing agents, such as gelatine and rosin, and/or inorganic fillers, typically calcium and magnesium carbonates, can be present depending on the original fibers and papermaking processes.^14,15^ Paper degradation, mainly caused by hydrolytic and oxidative reactions, can lead to the formation of low molecular weight degradation products, including organic acids, low molecular weight saccharides, and their oxidation, with the formation of carbonyl groups, the latter responsible for paper yellowing.^13,16^ However, the complexity of NIR spectra makes their direct interpretation by band assignment virtually impossible, and mathematical and statistical tools (chemometrics) are required to extract useful information.^17,18^

A wide variety of machine learning algorithms has been developed to address diverse data and problem types across the chemical sciences.^19−22^ Supervised machine learning (SML) methods have become a means of inferring the relationships/correlations between the property of interest and spectral features of complex, multi-component heritage materials, such as paper,^23−29^ parchment,^30,31^ plastics,^32^ wood,^33−36^ and leather.^37^ Previous studies combined infrared (IR) spectroscopy almost exclusively with Partial Least Squares (PLS) to explore dating applications for paper materials (Table S1). Some SML methods, such as least squares support vector machine^38^ and convolutional neural networks,^39^ have also been applied to IR data for date classification of modern paper (1940–1980), within a 5-year period. However, a controversial aspect of the predictions based on IR spectral data is the cause-effect relationship between the property of interest and the spectral features; i.e., the underlying process remains insufficiently clarified. In the literature, systematic analysis of the underlying process and associated errors in IR spectroscopy-based methods applied to heritage objects is scarce,^23^ leading to limited utility of such data. There is a need for a clearer understanding of not only model development and performance but also the uncertainties associated with the methods for dating.

We can hypothesize that dating through NIR spectroscopy-based methods is enabled by the well-known changes in the manufacturing processes of paper materials and the accumulation of degradation products due to natural aging, which are in turn reflected in an enormous complexity of spectral features. Great variability in paper composition can be found in historical collections.^14,15^ While the technology of rag paper production was almost homogenous from its invention until ca. 1850, the technology of machine-made paper, which was introduced in the early 19th century due to the high demand for paper and unavailability of traditional raw fiber materials (e.g., cotton, linen, and hemp rags), changed enormously and rapidly.^9^ Numerous compositional changes occurred during the period of relevance to our research (1850–2000) with the co-presence of rosin-sized paper, bleached paper, and quasi-neutral and alkaline paper with various cationic starching techniques. On the other hand, when dealing with historical objects, degradation cannot be ignored. Although the extent of degradation is related to age, paper can degrade to very different extents, depending on both material and environmental conditions.^40^ Influence of both paper variability (e.g., inorganic compounds) and degradation (e.g., cellulose oxidation and changes in crystallinity) have been found to be meaningful for the dating models developed in forensics using IR spectroscopic data of modern paper (1940–2012).^38,39,41,42^ However, the question to what extent degradation and compositional changes influence the date of real paper predicted by SML methods using NIR spectroscopic data remains.

To this end, we present a systematic study of the dating of a real book paper collection through analysis of NIR spectroscopic data using three SML methods, i.e., PLS, Random Forest (RF), and k-Nearest Neighbors (kNN). While we chose PLS, a parametric method applied in similar studies (Table S1), for comparison purposes, RF and kNN are tested as two alternative non-parametric techniques based, respectively, on tree-based methods and similarity measures, to explore their advantages and drawbacks in terms of both predictive and interpretative ability. To ensure that the NIR spectroscopy-based method using PLS, RF, and kNN applies to real and diverse documents, we analyzed 100 books from the general collection of the National and University Library of Slovenia (Ljubljana, Sl) with publication dates ranging from 1851 to 2000 (see Section S2.1 for details). In each book, NIR spectra were acquired on different pages, and at different points in each page to address both paper variability within the book block and degradation (see Section S2.2 for details). The same book, in fact, can be made of paper from different batches, and the outer margins are generally more degraded due to exogenous pollutants, oxygen, and light.^43^ We compare the three SML methods testing spectral preprocessing and variable selection methods. Different preprocessing strategies may yield comparable or different results in terms of model accuracy. However, the aim of this study is neither to provide the optimal generalizable model nor spectral preprocessing and variable selection methods that generally perform best, but to explore the underlying principles of such models, their accuracy, and whether and how factors associated with paper variability and degradation influence the resulting models. We demonstrate that the non-parametric techniques, i.e., RF and kNN, markedly increase predictive performance compared to PLS, although PLS, RF, and kNN assign similar levels of importance to the NIR spectra regions in the identification of the underlying process. We then evaluate the differences between subsets of spectra as a gauge of compositional changes and natural degradation, to understand whether and how such factors influence the uncertainty of the predicted date.

## Results and Discussion

### Preprocessing and Modeling

Spectra truncation was the first preprocessing step, as shown in the workflow of Figure S3. We removed the visible and first NIR range (up to 1000 nm) to avoid that information related to chromophores could affect the modeling, as well as the range from 2300 to 2500 nm due to its low signal-to-noise ratio. Moreover, since a strong band of O–H vibrations of H_2_O occurs at approximately 1930 nm,^44^ the 1900–2000 nm spectral range was further removed to reduce the influence of the moisture content of the paper, which varies according to the environmental conditions of storage. After truncation, we followed a data-driven approach to select the best spectral preprocessing technique and most informative variables according to the predictive ability of the resulting model (see Section S2.3.1 for details).

A good spectral preprocessing technique should remove the undesired sources of variability in the NIR data, including scattering, baseline shift, and noise, which can reduce the chances of successful correlation.^45^ As reported in Table S4, seven combinations of two commonly used algorithms were tested: Standard Normal Variate (SNV)^46^ and Savitzky–Golay (SG) algorithm^47^ with zero (SG0), first (SG1), and second (SG2) order derivatives and an 11-point smoothing window. Variable selection is based on the assumption that not all the variables (wavelengths) are either strongly related to the property of interest or carriers of non-redundant information.^48^

Removal of non-relevant and redundant variables can lead not only to more precise and accurate regression models but also to more parsimonious models, useful for an easier interpretation of the underlying process that generated the data.^49−51^ Two variable selection methods were tested: Boruta,^52^ a variable selection wrapper algorithm that uses RF, and Genetic Algorithm (GA),^53,54^ which, inspired by the principles of natural selection and genetics, evaluates candidate solutions (combinations of variables) using a fitness function in an iterative process in search of the suboptimal combination of variables, i.e., with the highest fitness value. For GA optimization, the fitness values corresponding to the prediction ability of PLS, RF, and kNN were compared, thus we have three GA-based variants, i.e., PLS-GA, RF-GA, and kNN-GA, respectively (see Section S2.3.1 and Table S5 for details).

The prediction accuracy of the models was assessed using root mean square error of repeated 10-fold cross-validation with 100 iterations (RMSE_CV100_), the corresponding standard deviation (SD) and the 95% confidence interval (CI95%) (Tables S8–S10).

**Figure 1:**
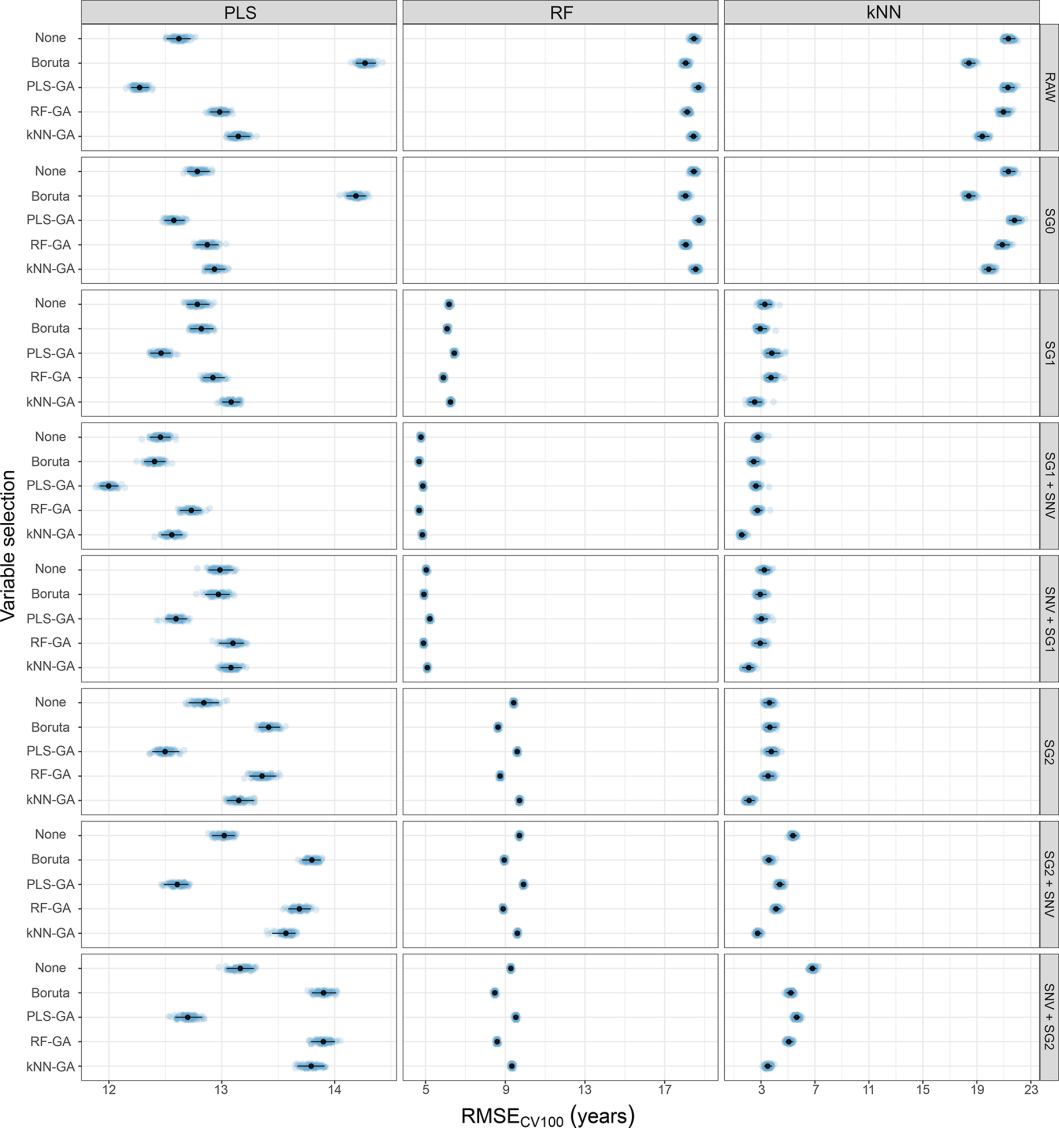
Model performance as results of spectral preprocessing and variable selection methods using all spectra. Each panel of the grid reports the distribution of performance in terms of RMSE_CV100_ of Partial Least Squares (PLS), Random Forest (RF), and k-Nearest Neighbors (kNN), by column, using the four subsets of variables obtained through the variable selection methods (Boruta, PLS-GA, RF-GA, and kNN-GA), as well as the whole set of variables (None), considering the different data preprocessing adopted, by row. In particular, the errors of the repetitions are projected through blue points, their average is indicated with a black point, whilst the black line shows the 95% confidence interval.

The spectral preprocessing and variable selection methods have different effects depending on the SML methods used, as shown in Figure 1. Although to different extents, the accuracy of RF and kNN is substantially improved by first and second-order derivatives. SG1 with SNV provides the most accurate results using PLS, RF, and kNN. As it has been proven in some other applications,^25,41^ variable selection methods increase the potential to obtain more accurate predictions while relying on a much smaller number of variables. The best variant for GA depends on the SML method involved. PLS-GA, RF-GA, and kNN-GA lead to the lowest RMSE_CV100_ for PLS, RF, and kNN, respectively, although especially for RF other variants lead to comparable results, considering the confidence intervals. While GA and Boruta can provide similar improvements in view of the predictive ability (Tables S8–S10), they differ in terms of parsimony (Table S11). All GA-based variants pick about 50% of the number of variables, while Boruta selects less effectively (∼85%) the variables preprocessed by the first derivative (i.e., SG1, SG1 + SNV, SNV + SG1).

**Figure 2:**
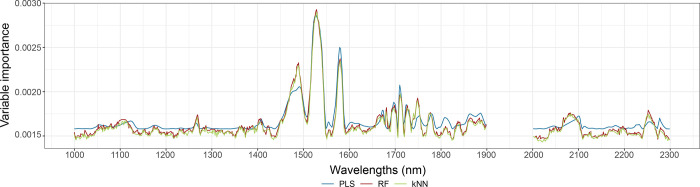
Variable importance for PLS (blue), RF (red), and kNN (green) models developed using all the spectra.

For each SML method, the two best models in terms of prediction accuracy were compared using two-tailed t-test (Table S12). For PLS and kNN, significant differences were found, thus for PLS and kNN we chose the model preprocessed by SG1 + SNV with PLS-GA, and SG1 + SNV with kNN-GA, respectively. Whereas for RF we chose the model developed using RF-GA as it requires less variables than Boruta to achieve comparable accuracy. RF and kNN, with RMSE_CV100_ of ca. 5 and 2 years, respectively, show higher predictive ability compared to PLS, with RMSE_CV100_ of ca. 12 years. Although based on different approaches, the two non-parametric methods perform much better than PLS (Figure S11). However, to explore the underlying process for each SML method, we evaluated the importance assigned by the SML methods to each variable (wavelength) among those selected. To compare all SML methods with the same measure of variable importance, we measured how much the inverse of the error score increases with models built using one variable each time. Interestingly, Figure 2 shows that the same spectral ranges result to be informative regardless of whether we use PLS, RF, or kNN, as confirmed by the high correlation coefficients (Table S13) obtained by comparing the variable importance of the common wavelengths selected. The most important wavelengths are in the range roughly from 1490 to 1580 nm, and from 1700 to 1780 nm, which can be associated with C–H and O–H stretching first overtones, typical of the cellulose structure, and N–H stretching first overtone from amides/proteins.^44,55^ The most informative variables used to build the dating models are common to the three SML methods, suggesting that the underlying process robustly generated the predicted dates.

### Influence of Age and Sampling Methods

To explore the possible influence of the age of books on the dating models, the books were divided into three subsets based on their publication date, i.e., 1851–1900, 1901–1950, and 1951–2000. On the other hand, to investigate whether the sampling method, in terms of both paper variability within the book block and degradation, influences the dating models, NIR spectra were acquired in the front, middle, and back pages of the book block, and in the gutter, center, and outer margin of each page (see Section S2.2). The spectra were thus divided into subsets based on the pages and points on the page where the measurement was made. Table 1 reports the groups of spectra by “Publication date”, “Page” and “Point”, and the corresponding subsets.

**Table 1:** Groups of “All”, “Publication date”, “Page” and “Point” Spectra^a^

groups	subsets	*n*_b_	*n*_s_
All		100	3000
Publication date	1851–1900	34	1020
	1901–1950	32	960
	1951–2000	34	1020
Page	Front	100	900
	Middle	100	1200
	Back	100	900
Point	Gutter	100	1000
	Center	100	1000
	Margin	100	1000

a For each group, except for “All”, spectra are further divided into three subsets based on a 50-year time period for the publication date, and on where spectra were acquired depending on the pages of the book block and the point of the page. The corresponding number of books (*n*_b_) and spectra (*n*_s_) are reported.

Moreover, in order to explore the trend of prediction accuracy with an increased number of spectra in the groups from 50 up to 900, a simulation study was designed (see Section S2.3.2). Tables S14–S16 report RMSE_CV100_, SD, and CI95% for each subset of the “Publication date” group using PLS, RF, and kNN. It is to be noted that the variability of prediction in the group by “Publication date” is restricted over a period of 50 years, unlike the other groups which range over a period of 150 years.

**Figure 3:**
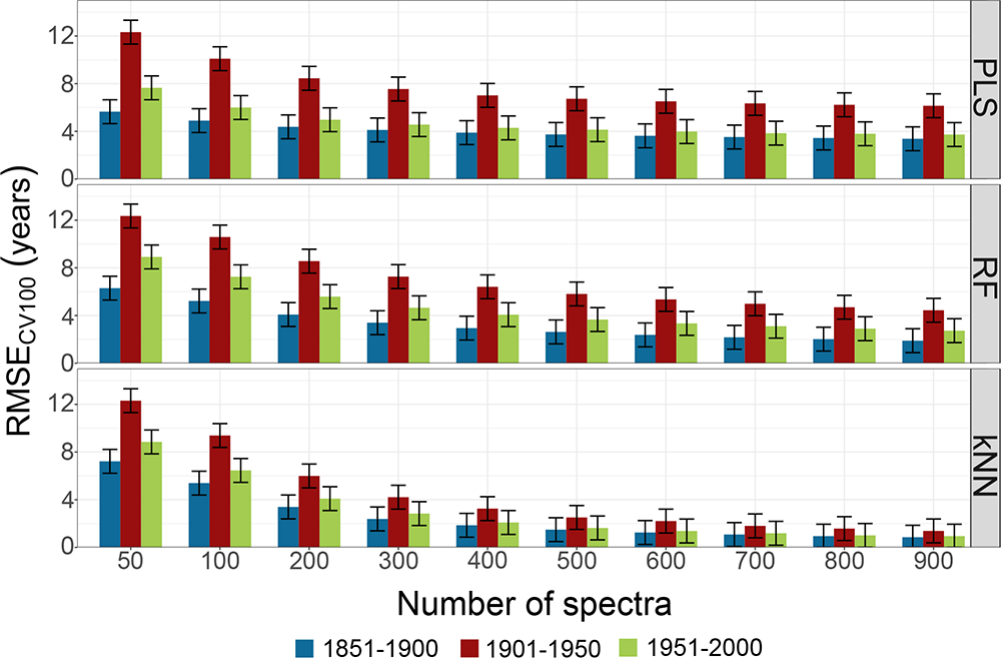
RMSECV_100_ of PLS, RF and kNN according to the number of spectra for the subsets of the “Publication date” group. The error bars represent 1 year.

Figure 3 shows RMSE_CV100_ computed for the subsets of the “Publication date” group with error bars of 1 year. For a meaningful interpretation of the results, only differences higher than 1 year should be considered relevant, that is the accuracy of our reference data, as the publication year can either refer to January or December.

Using a small number of spectra, the predictions obtained from PLS, RF, and kNN for the books in the “1901–1950” subset result to be the least accurate ones if compared to the “1851–1900” and “1951–2000” subsets. Previous studies reported different accuracies in dating Chinese^24^ and European^29^ paper due to the doubtful quality of reference data for most pre-1900 samples, and to the small sample size for pre-1850 paper, respectively (Table S1). Here, with comparable accuracy of reference data and sample size, the books of the “1901–1950” subset appear to be the most challenging. Several factors, such as provenance, extent of degradation, and past storage environmental conditions, may play a role in this difference. However, a reasonable explanation can be traced back to the exceptionally high variability in paper composition occurring from the end of the 19th century and the first half of the 20th century. A high variability in paper composition may lead to higher uncertainty in dating models when different paper types correspond to the same publication dates. Changes in the manufacturing practices and raw materials used characterized this transitional and experimental period of papermaking.^15^ Rag fibers, including cotton, linen, and hemp, were mixed in different ratios to wood fibers, which could be produced directly from wood by thermomechanical processes (groundwood fibers) or by partial delignification using various bleaching methods (bleached fibers).^15^ We account for paper variability using a stratified random selection of the books to be analyzed and by measuring different pages through the book blocks (see Sections S2.1 and S2.2 for details). However, this may not have included all types of paper from the period of interest. This can lead to discrepancies when testing model performance using independent test sets, indicating that more paper types should be included in the future.

**Figure 4:**
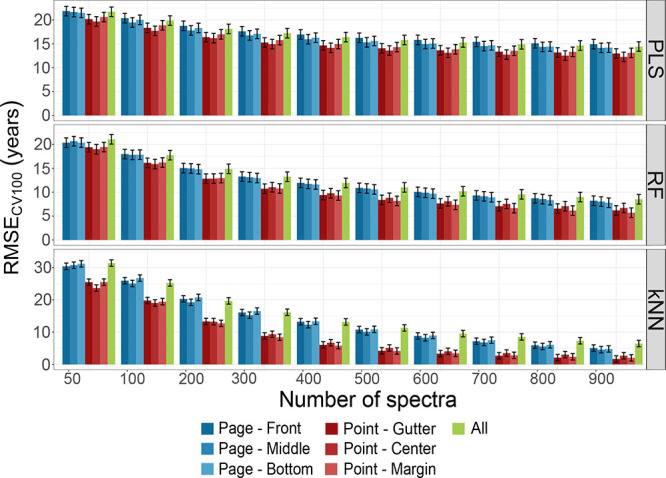
RMSE_CV100_ of PLS, RF, and kNN according to the number of spectra. The bars represent the models developed using the subsets of the “Page” (blues) and “Point” (reds) groups, and the “All” (green) group. The error bars represent 1 year.

Figure 4 shows the RMSE_CV100_ calculated for each subset of spectra for the “Page” and “Point” groups, with the error bars of 1 year.

As expected, the model performance improves as the number of spectra increases, although to different extents: while PLS errors decrease very gradually, those associated with RF and kNN decrease rapidly, especially for kNN. The models developed using all the spectra (Table S17), without distinction of pages and points of measurement, generally lead to higher RMSE_CV100_ compared to the models developed on the subsets of spectra related to pages or points, regardless of the SML method employed (Tables S18–S23). For all three SML methods, RMSE_CV100_ for subsets within the same group is generally similar regardless of the number of spectra included. Using kNN, the models developed for the “Point” group, i.e., the spectra acquired on different pages but only at one measurement point (either gutter, center, or margin), lead to better performances than those developed using the “Page” group, i.e., the spectra acquired at different points but only on the front, middle or back pages of the book block (Tables S20 and S23). Thus, acquiring spectra on different pages of the same book, which can reflect paper variability within the book block, is beneficial, especially for kNN.

Table 2 reports RMSE_CV100_ and the corresponding SD of the models developed using the maximum number of the available spectra for the “Point” group (i.e., 1000) and for the “All” group (i.e., 3000), as well as 1000 spectra for the “All” group, for the sake of comparison.

**Table 2:** Summary of RMSE_CV100_ and the Corresponding SD for PLS, RF, and kNN Built on the Subsets of the “Point” and “All” Groups^a^

groups	subsets	*n*_s_	PLS	RF	kNN
			RMSECV_100_ ± SD (year)	RMSECV_100_ ± SD (year)	RMSECV_100_ ± SD (year)
Point	Gutter	1000	12.81 ± 0.10	5.89 ± 0.08	1.32 ± 0.30
	Center	1000	12.07 ± 0.10	6.36 ± 0.06	2.37 ± 0.20
	Margin	1000	12.94 ± 0.11	5.36 ± 0.08	1.65 ± 0.26
All		1000	14.24 ± 0.35	8.10 ± 0.17	5.89 ± 0.69
		3000	12.00 ± 0.04	4.68 ± 0.04	1.57 ± 0.13

a The number of spectra (*n*_s_) used to build the models is reported.

**Figure 5:**
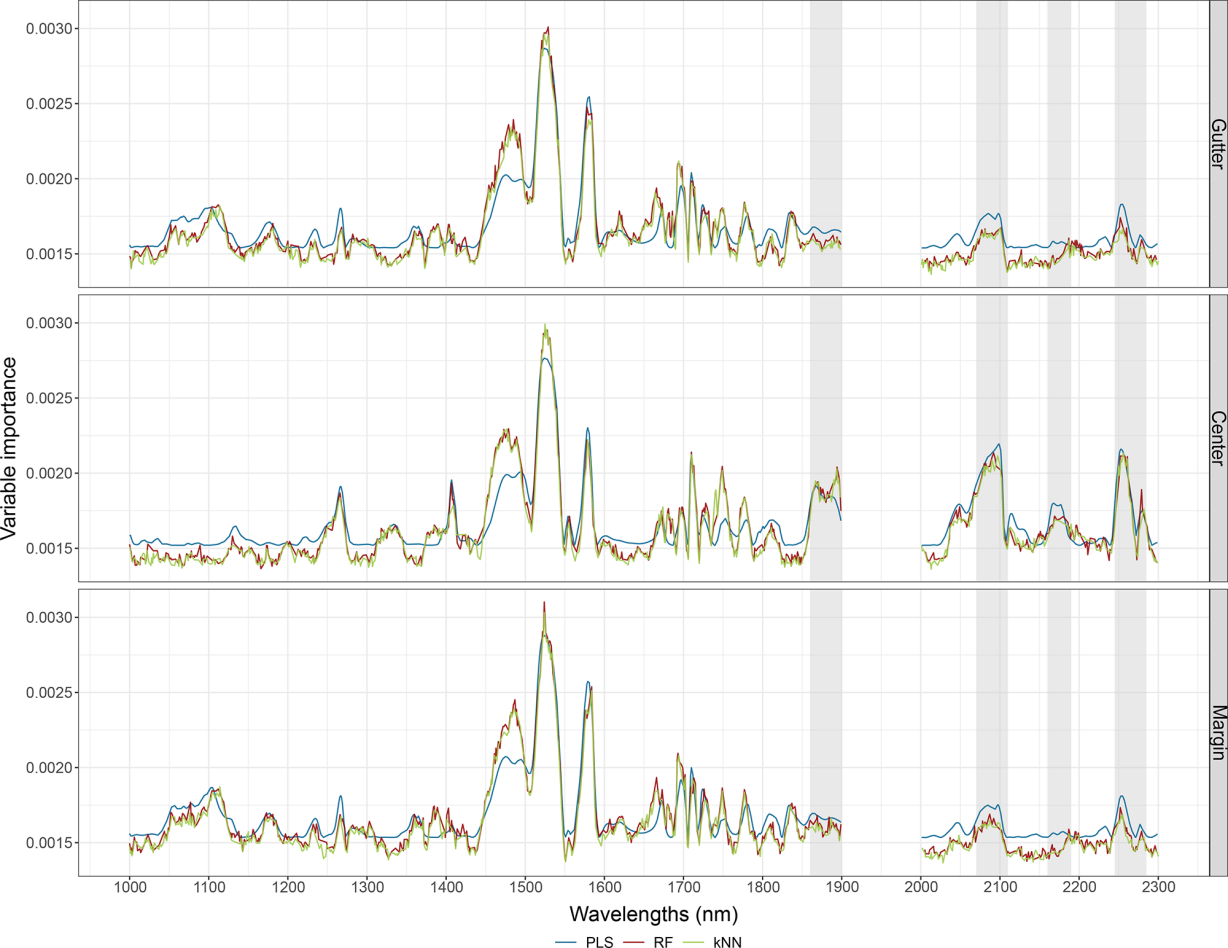
Variable importance for PLS (blue), RF (red), and kNN (green) developed on the subsets of the “Point” group. The gray areas highlight the spectral regions, which differ mostly comparing the subsets.

For each SML method, no meaningful difference (i.e., higher than 1 year) can be highlighted between the models developed using spectra acquired on the gutter, center, or margin of the page. As shown in Figure 5, we confirm that PLS, RF, and kNN give similar levels of importance to the same spectral ranges even when we use subsets of spectra for the “Point” group (Table S24). However, Figure 5 shows differences in terms of the most important variables, especially at >1850 nm, among models developed using “Gutter”, “Center” and “Margin” subsets. Specifically, for the models developed on the “Center” subset: the band at ca. 1870 nm can be associated with C–Cl stretching overtone from chlorinated hydrocarbons,^44,55^ as residual of Cl-based bleaching methods in papermaking; the three broad absorption bands at roughly 2100, 2180, and 2280 nm can be associated with a combination of primary amides, including N–H stretching, N–H bending second overtone, and C–H bending second overtone from amides/proteins.^44,55^ These latter bands have been reported as important wavelengths related to gelatine content in paper.^25^ Gelatine, derived from collagen, i.e., the connective tissue in skin and ossein of animals, was widely used as a sizing material from the 14th to the 19th century. Although the protein content gradually decreased toward the early 19th century, when alum-rosin size became widely used, protein was found in paper dated after 1850.^14,56^ Since Scheele’s discovery of chlorine in 1774,^9^ various Cl-based methods were experimented to bleach paper^57^ up to the current and more sustainable elemental chlorine-free bleaching.^58^ This confirms that compositional changes, ascribable in this case to chlorine and protein presence in paper, may play a role in predicting the date using NIR spectral features.

On the other hand, the gutter of the page is generally less degraded than the margin, as the latter is exposed to pollutants, oxygen and light,^43^ which promote degradation. However, very similar spectral ranges have the same level of importance for models using the “Gutter” and “Margin” subsets. Additionally, since the center of a page is expected to be less degraded than the gutter, we can reasonably assume that the differences between the models built using the “Gutter” and “Center” subsets are related to the different backgrounds used for measuring the spectra, i.e., a stack of paper and the white reference target in the gutter and the center, respectively (see Section S2.2 for details). The penetration depth of NIR radiation is about 1–3 mm,^59^ meaning that more layers provide a higher signal-to-noise ratio. Therefore, intuitively more layers are better, and as found in previous studies,^24,29^ 4–5 layers are sufficient. However, considering the variability in terms of natural degradation of a real book collection and heterogeneity of paper within a stack, 1 layer measured at the center with white reference as background provides spectral features related to paper composition aspects, with higher importance than multiple layers at the gutter or margin, as shown in Figure 5.

Moreover, the predictions based on the “Center” subset are more robust in terms of model transferability because the center of the page is less affected by the environmental conditions, which can differ between different storage environments. Thus, such models can be applied to collections that have experienced different environmental conditions. Therefore, the results of the models developed using 1000 spectra acquired at the center of the pages (Table 2, “Center” subset) were used for comparison with the literature data. Since other studies analyzed different date ranges (Table S1), the normalized root mean squared error of cross-validation (NRMSE_CV100_) is also reported and is computed by eq 1:
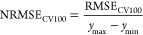
1where *y*_max_ and *y*_min_ are the maximum and minimum reference values for the property of interest (i.e., 2000 and 1851 in this study), respectively. We found an accuracy of 12 years for PLS, corresponding to NRMSE_CV100_ of 0.08. This result lies between those already reported in the literature using PLS for post-1851 European paper,^29^ i.e., 0.06, and those reported for Chinese^24^ and modern^42^ paper, ranging from 0.13 to 0.15. However, as discussed above, the NRMSE_CV100_ decreases to 0.04 and 0.02 using RF and kNN, respectively. These values correspond to approximately 6 and 2 years, over the time period 1851–2000, that are the lowest accuracies so far reported for dating of paper from a real collection.

### Uncertainty Evaluation

We employed three SML methods, which markedly differ in terms of flexibility, kNN being the most flexible.^60^ For an in-depth investigation of the accuracy and precision, we decompose the bias-variance trade-off characterizing the mean square error (MSE)^60^ as shown in eq 2:
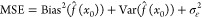
2where Var((*x*_0_)) refers to the amount by which , i.e., the function that approximates the true function, would change if we estimated it using different datasets, Bias^2^((*x*_0_)) refers to the squared difference between  and the true function *f*, and σ_ε_^2^ is the variance associated with the error term ϵ, which may include unmeasured variables and unmeasurable variation.^60^ By definition, ϵ cannot be predicted using the input variables. It is known as irreducible error because it does not depend on how we approximate . Whereas, the sum of Var((*x*_0_)) and Bias^2^((*x*_0_)) represents the reducible error, which reflects the performance of  in estimating the true function *f*.^60^ Therefore, to compare the ability of the three SML methods to minimize the reducible error, we assess the decomposition of 100-repeated 10-fold cross-validation error (MSE_CV100_) into variance and squared bias.

**Figure 6:**
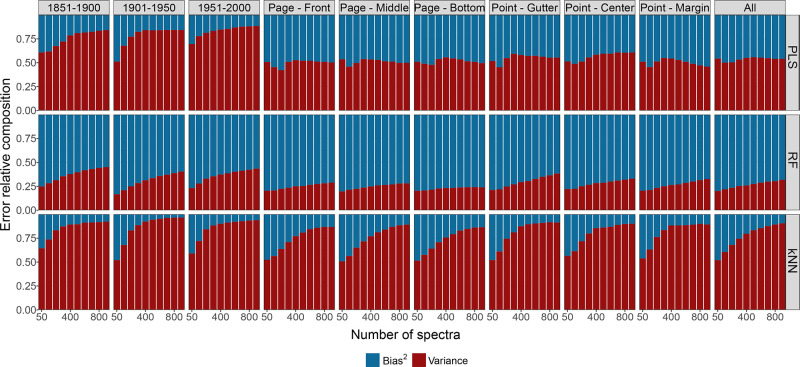
Decomposition of reducible error into relative contributions of squared bias (blue) and variance (red) with an increased number of spectra. Each panel of the grid reports the relative contributions for the three SML methods (by row) and for the combined subsets of spectra (by column).

Figure 6 shows the relative contributions of variance and squared bias according to the number of spectra from the simulation study for each subset of the groups (Tables S25–S27). For PLS, the decomposition highlights approximately a constant pattern along all the number of spectra for all subsets, where variance and squared bias have the same contribution, except for the “Publication date” group. In the latter group, the variance contribution is higher than the squared bias and increases with the number of spectra. For RF and kNN, the larger the number of spectra, the higher the variance contribution; the evident difference being that for RF, the squared bias always represents a higher contribution to the reducible error, while for kNN this is mostly due to the variance. These results show the high capability of RF to reduce the variance component of the reducible error because of the de-correlated tree-based structure, while the high flexibility of kNN leads to a larger variance. Therefore, the instrumentation, such as spectral resolution, and the measurement procedure, such as sample preparation, contribute mostly to the model performance using kNN.

## Conclusions

In summary, our study demonstrated that the best dating accuracy of as much as 2 years is achieved with the non-parametric methods, although they share the same underlying processes that generate the data with the parametric method. The methods do not depend on degradation as we have shown that the models developed on more degraded areas do not meaningfully differ from those developed on less degraded areas. This ensures the applicability to collections, historically stored in different environmental conditions. Future study should include testing of the model performances using independent test sets from different libraries and regions from the same period (1851–2000). While we randomly selected the books to have a representative sample set, some types of paper may not have been included in this selection and may need to be in the future, therefore. Our results should encourage a systematic investigation of the underlying principles for dating, as well as the determination of material properties of paper and other heritage materials of organic origin, using IR spectroscopy and machine learning. This would increase their practical applicability to collections in heritage institutions.

## Supporting Information Available

Literature data, experimental details related to samples and sample strategy, NIR spectroscopy and data analysis, additional figures and tables of the results of the models (PDF)
Datasets including metadata of the books analyzed, raw spectra, and spectra naming convention (ZIP)

## Notes

*Data and code availability.* The datasets generated during the current study are available from the Supporting Information files. The codes generated are available from the corresponding author upon reasonable request.
