# Supplementary material for: Near-Infrared Spectroscopy and Machine Learning for Accurate Dating of Historical Books
Source: J Am Chem Soc. 2023 May 22;145(22):12305–14. doi: 10.1021/jacs.3c02835 (PMC10251519; doi:10.1021/jacs.3c02835)
Supplement: Supplementary file 1 — ja3c02835_si_001.pdf [file ja3c02835_si_001.pdf]

Supporting Information

Near-Infrared Spectroscopy and Machine Learning for Accurate Dating of Historical Books

Floriana Coppola*,§, Luca Frigau†, Jernej Markelj§, Jasna Malešič#, Claudio Conversano†, and Matija Strlič§,‖

§ Faculty of Chemistry and Chemical Technology, University of Ljubljana, Večna pot 113, Ljubljana 1000, Slovenia

† Department of Business and Economics, University of Cagliari, Via Sant'Ignazio da Laconi 17, Cagliari 09123, Italy

National and University Library of Slovenia, Turjaška ulica 1, Ljubljana 1000, Slovenia

‖ Institute for Sustainable Heritage, University College London, 14 Upper Woburn Place, London WC1H 0NN, UK

*Correspondence to: floriana.coppola@fkkt.uni-lj.si

Table of Contents

S1 Literature data S1
S2 Experimental Section S2
S2.1 Samples and Sampling Strategy S2
S2.2 NIR Spectroscopy S3
S2.3 Data Analysis S5
S2.3.1 Preprocessing S6
S2.3.2 Simulation Study S14
S3 Supplementary Figure S15
S4 Supplementary Tables S16
S5 Supplementary References S26

S1 Literature data

Table S1 reports the results of previous studies to date paper using mid-IR and NIR spectral data combined with PLS. In order to easily compare the reported results, since different date ranges were analyzed, the Normalized Root Mean Square Error of Prediction (NRMSEP) is also reported, and computed as follows: $\text{NRMSEP} = \frac{\text{RMSEP}}{y_{\max} - y_{\min}}$, where RMSEP is the Root Mean Square Error of Prediction, $y_{\max}$ and $y_{\min}$ are, respectively, the maximum and minimum reference values in the property of interest (i.e., date).

Table S1. Summary of the data reported in the literature on the use of mid-IR and NIR spectral data to date paper by PLS. The spectral preprocessing algorithms used are reported, i.e., Savitzky-Golay (SG), Multivariate Scatter Correction (MSC), Standard Normal Variate (SNV), Orthogonal Signal Correction (OSC) and Generalized Least Squares Weighting (GLSW). The performance of the models was reported in terms of Root Mean Square Error of Prediction ($RMSEP$), the corresponding Normalised Root Mean Square Error of Prediction ($NRMSEP$) and coefficient of determination (R^2).

<table>
<tr><th>Date range</th><th>Spectral range (nm)</th><th>Spectral pre-processing algorithm</th><th>Variable selection</th><th>Machine learning method</th><th>RMSEP (year)</th><th>NRMSEP</th><th>R^2</th><th>Ref.</th></tr>
<tr><td>1650 – 1850</td><td rowspan="2">1538 – 10000</td><td rowspan="2">None</td><td rowspan="2">None</td><td rowspan="2">PLS</td><td>8.6</td><td>0.04</td><td>0.89</td><td rowspan="2">[1]</td></tr>
<tr><td>1851 – 2005</td><td>8.6</td><td>0.06</td><td>0.94</td></tr>
<tr><td>1799 – 1990</td><td rowspan="2">1600 – 2400</td><td>SNV, MSC,</td><td rowspan="2">None</td><td rowspan="2">PLS</td><td>24</td><td>0.13</td><td>0.53</td><td rowspan="2">[2]</td></tr>
<tr><td>1900 – 1990</td><td>SNV, MSC, SG[a]</td><td>13</td><td>0.14</td><td>0.41</td></tr>
<tr><td rowspan="4">1985 – 2012</td><td rowspan="4">2500 – 4082
4474 – 15385</td><td>SNV, SG[b], mean-centering</td><td>None</td><td>PLS</td><td>3.8</td><td>0.14</td><td>0.90</td><td rowspan="4">[3]</td></tr>
<tr><td>SNV, SG[b], OSC, mean-centering</td><td>None</td><td>PLS</td><td>4.0</td><td>0.15</td><td>0.89</td></tr>
<tr><td>SNV, smoothing GLSW, mean-centering</td><td>None</td><td>PLS</td><td>3.6</td><td>0.13</td><td>0.91</td></tr>
<tr><td>SNV, SG[b], mean-centering</td><td>sparse PLS</td><td>sparse PLS</td><td>4.0</td><td>0.15</td><td>0.88</td></tr>
</table>

[a]First derivative, second order polynomial and 3-point window smoothing
[b]Second order polynomial, 21-point window smoothing

S2 Experimental Section

S2.1 Samples and Sampling Strategy

The analyses were designed to explore the underlying process in dating models provided by SML methods using NIR spectroscopic data, as well as the possible sources of uncertainty associated with the publication dates of the books and the sampling method.

The books analyzed are from the general collection of the National and University Library of Slovenia (NUK), and are housed in two different storage locations, both in Ljubljana (Slovenia). The Library holds approximately 2.8 million records, including the main national collection, a collection of scientific literature, and a collection of special library materials[4]. The books from the monographic book collection published between 1851 and 2000, when largest compositional changes in paper technology occurred, were of interest to our case study. Our reference data, i.e., the publication years reported on the books, have an accuracy of ± 1 year since a book could be published from January to December, i.e. within the span of one year.

To have a sample set representative of the period 1851-2000 a stratified sampling strategy was designed with the decade of publication as the criterion for stratification. A total of 100 books was analyzed. Table S2 reports the number of books analyzed per decade.

Table S2. Sample sizes as the number of items (books) analyzed in each stratum (decade) of publication date.

Stratum of publication date	Sample size
1851-1860	6
1861-1870	6
1871-1880	7
1881-1890	7
1891-1900	7
1901-1910	7
1911-1920	7
1921-1930	6
1931-1940	6
1941-1950	7
1951-1960	7
1961-1970	7
1971-1980	7
1981-1990	7
1991-2000	6

The books were randomly selected from within a decade, and Figure S1 shows the number of randomly selected samples per publication year. Metadata including bibliographic information (e.g., title, author and publication year) were recorded and are reported in Supporting Information file (Dataset_S1).

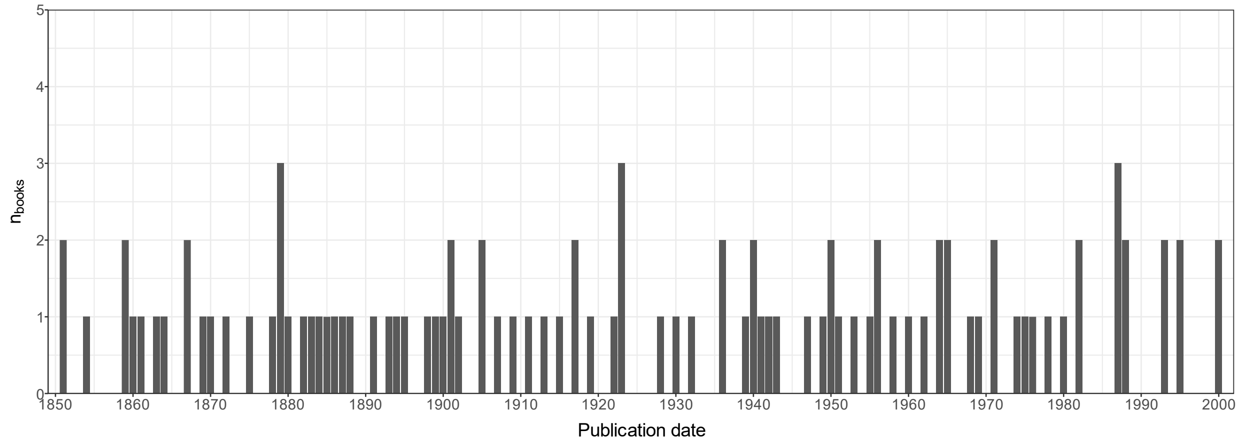

Figure S1. Number of books (n_{books}) randomly selected per publication year.

S2.2 NIR Spectroscopy

Diffuse reflectance spectra were acquired in the range of 350-2500 nm using a portable UV-VIS-NIR ASD LabSpec® 5000 spectrometer (Malvern Panalytical Ltd, UK), equipped with a built-in light source, and three separate detectors: a 512-element silicon photo-diode array detector for the spectral interval 350-1000 nm, and two TE-cooled, extended range InGaAs photo-diodes for spectral intervals 1000–1800 nm and 1800–2500 nm. The sampling interval was 1 nm, while the spectral resolutions were 3 and 10 nm in the interval 350-1000 and 1000-2500 nm, respectively. Spectra, each an average of 200 scans, were acquired using a fiber-optic probe (Malvern Panalytical Ltd, UK) with spot diameter of approximately 2 mm in close contact with the samples, using Indico™ Pro software, version 6.0.3 (Analytical Spectral Device, USA). Splice correction for the light source was used to achieve a continuous spectrum. An ASD Spectralon® reference target (Malvern Panalytical Ltd, UK) was used for baseline measurement. In each book, 10 different pages were measured: 3 pages in the front, 4 pages of the middle and 3 pages at the back of the book block. On each page, 3 different points were measured: gutter, center and outer margin of the page (see Figure S2). Thus, a total of 30 spectra were taken for each book (taking about 30 min) in an area without ink and visible signs of localized degradation (e.g., foxing).

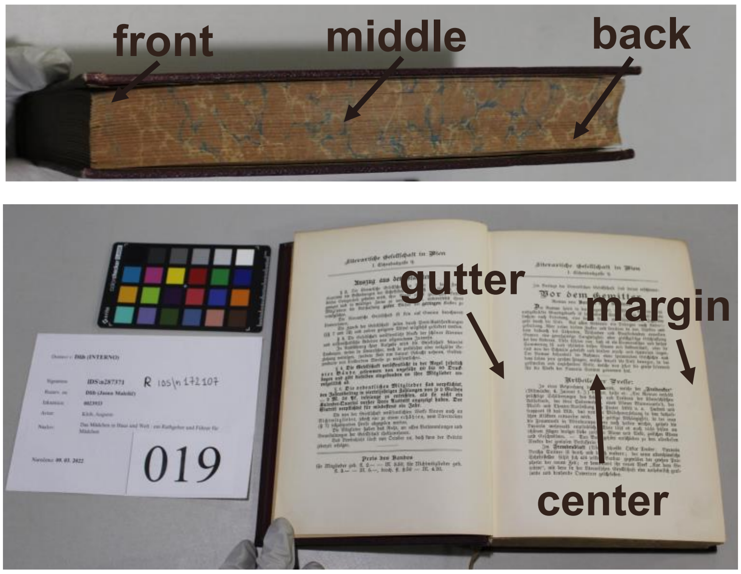

Figure S2. Illustration of where the spectra were measured. Front, middle and back pages of the book block (up), and gutter, center and outer margin of each page (down). The book (ID sample 019) in the picture is "Letzte Wiener Spaziergänge" by Von Daniel Spitzer, publication date 1894.

The stack of paper below the measured page was used as the background for the spectra acquired in the inner (gutter) and outer margins, while a Spectralon® reference target was used for the spectra acquired in the center of the page to avoid interferences due to the ink from the pages below. The penetration depth of NIR radiation in a matrix of organic substances is typically 1-3 mm[5], and it has been previously estimated that information is returned from up to 4-5 layers of purified cellulose sheets (approximately 0.5 mm)[1]. Therefore, the spectra can be considered as representative of both surface and bulk properties. All raw spectra were visually inspected, no outlier was detected. Some spectra exhibit different spectral features in some books, they were not considered to be outlier as they can express variability associated with different kinds of paper in the same book block or with extent of degradation.

To see if there is any influence associated with the compositional changes and sampling method, thus pages and points on the page analyzed, each spectrum was treated as an independent observation as it is representative of a unique combination of page and point where the measurement was made.

Spectroscopic analyses were conducted in a repository room of the NUK Library, where all the books were assembled at least two weeks before the analyses to let the paper acclimatize to the well-controlled environmental conditions. Temperature (19.0 ± 0.5 °C) and relative humidity (52 ± 1%) were measured using a Hobo MX100 datalogger (Onset Computer Corporation, USA).

The raw spectra are reported in Supporting Information files (Dataset_S2-S4), including the naming convention adopted for the spectra.

S2.3 Data Analysis

The workflow shown in Figure S3 illustrates the main steps of data analysis.

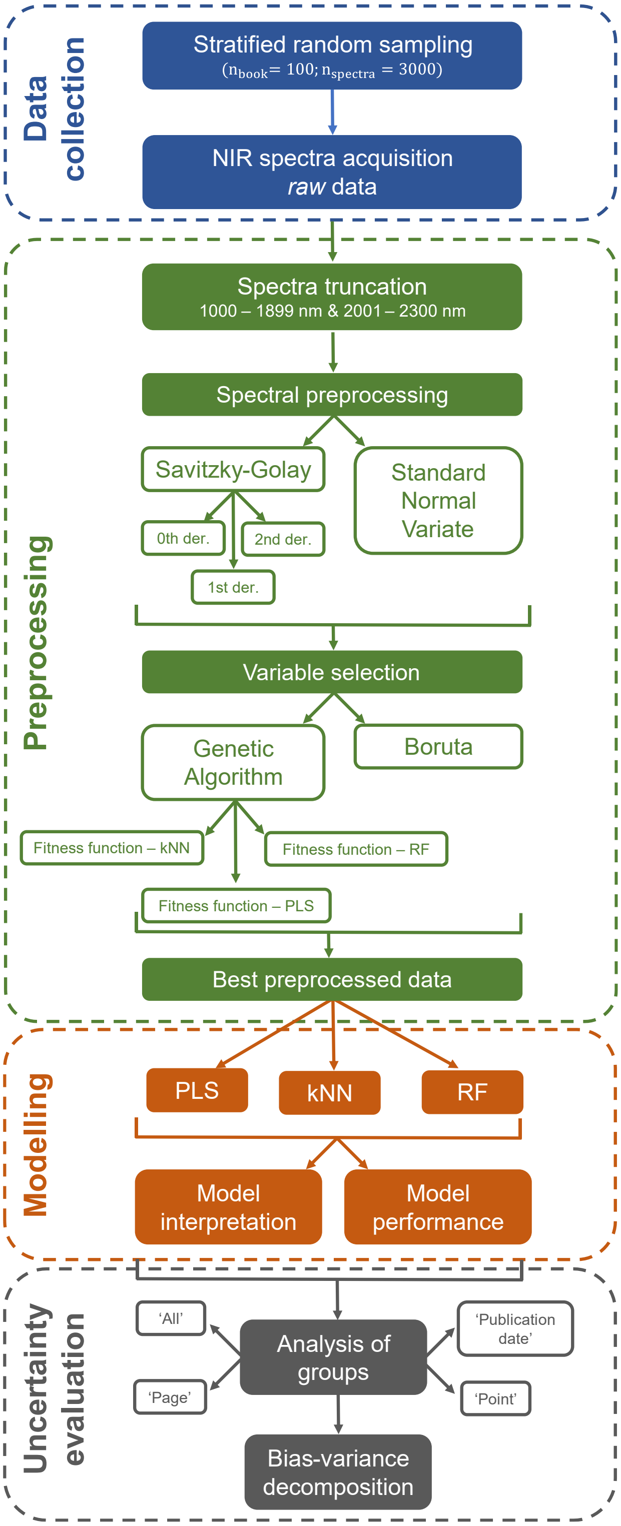

Figure S3. Data analysis workflow.

Preprocessing steps and SML models were conducted using R (vers. 4.2.1). Table S3 provides a list of the main R packages and their application in this study. All experiments were executed on individual Intel(R) Core(TM) i5-8500 CPU @ 3.00GHz processors, installed RAM 16.0 GB, using Windows 10 Home (vers. 21H2).

Table S3. R packages used in this study for spectral processing, variable selection and SML models.

Packages	Version	Reference	Application
tidyverse	1.3.2	[6]	Data manipulation and plot
prospectr	0.2.5	[7]	Spectral preprocessing
GA	3.2.2	[8]	Variable selection – GA
Boruta	7.0.0	[9]	Variable selection – RF
pls	2.8-1	[10]	PLS
ranger	0.14.1	[11]	RF
mlr	2.19.0	[12]	kNN

S2.3.1 Preprocessing

Table S4 reports the seven combinations we tested of two commonly used algorithms: Standard Normal Variate (SNV),[13] which performs a normalization of the spectra by subtracting each spectrum by its own mean and dividing it by its own standard deviation, and Savitzky–Golay (SG) algorithm,[14] a smoothing-based derivatization method.

Table S4. Summary of the parameter's settings of the spectral preprocessing algorithms implemented, including their abbreviations.

Abbreviation	Pre-processing algorithm and parameters settings
SG0	Savitzky-Golay (2nd order polynomial, 0th derivative, 11-point window)
SG1	Savitzky-Golay (2nd order polynomial, 1st derivative, 11-point window)
SG1 + SNV	Savitzky-Golay (2nd order polynomial, 1st derivative, 11-point window) + Standard Normal Variate
SNV + SG1	Standard Normal Variate + Savitzky-Golay (2nd order polynomial, 1st derivative, 11-point window)
SG2	Savitzky-Golay (2nd order polynomial, 2nd derivative, 11-point window)
SG2 + SNV	Savitzky-Golay (2nd order polynomial, 2nd derivative, 11-point window) + Standard Normal Variate
SNV + SG2	Standard Normal Variate + Savitzky-Golay (2nd order polynomial, 2nd derivative, 11-point window)

Figure S4 shows the truncated reflectance spectra (1000 – 1899 nm and 2001 – 2300 nm) of two books as collected by the LabSpec 5000 (raw spectra), and as preprocessed by the preprocessing algorithms.

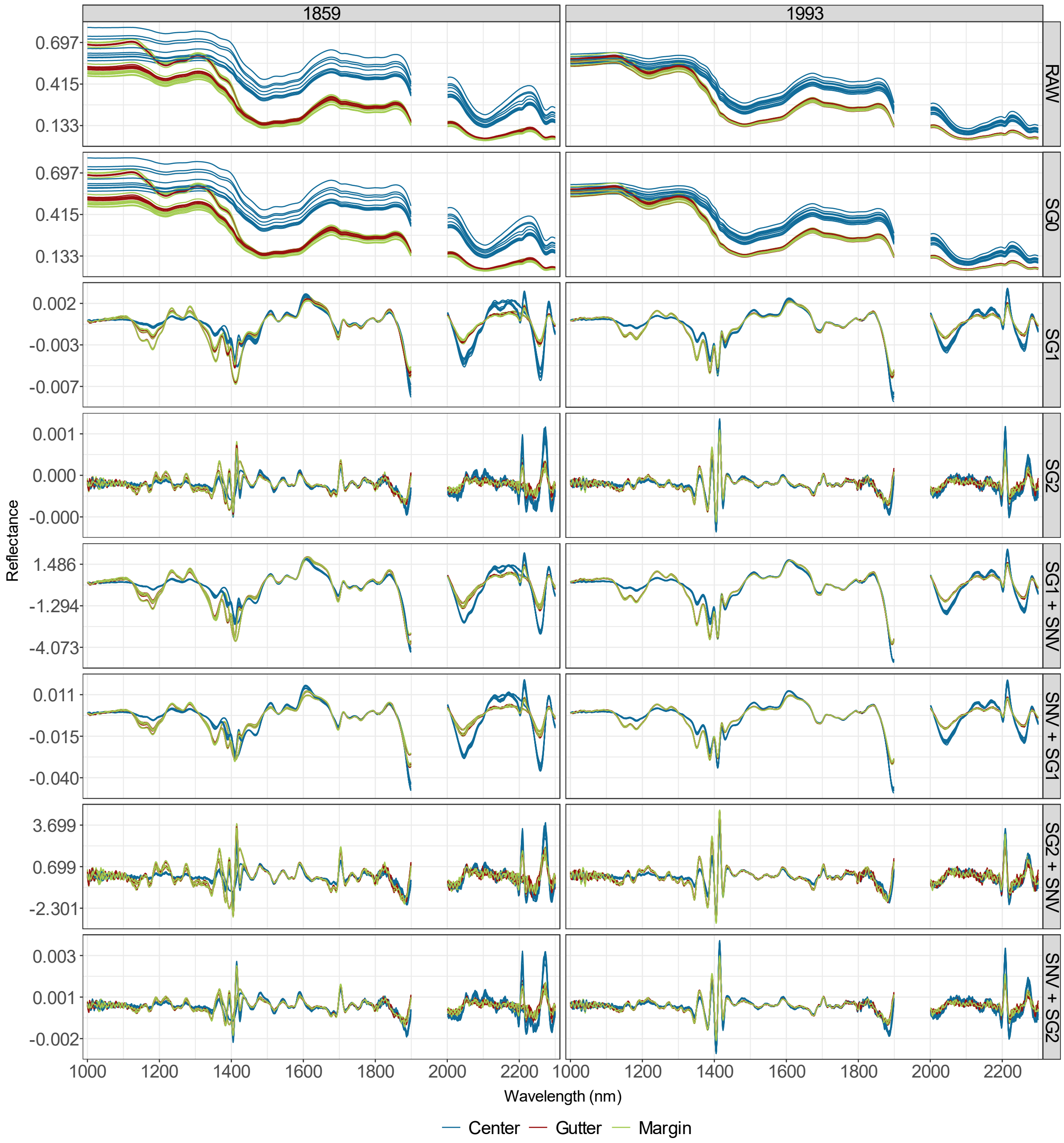

Figure S4. Truncated spectra of two books dated (left) 1859 and (right) 1993. By rows, each panel reports the spectra as collected by the UV-VIS-NIR LabSpec 5000 spectrometer (RAW), and as processed by 2nd order polynomial, 0th derivative, 11-point window Savitzky-Golay (SG0), 2nd order polynomial, 1st derivative, 11-point window Savitzky-Golay (SG1), 2nd order polynomial, 1st derivative, 11-point window Savitzky-Golay + Standard Normal Variate (SG1 + SNV), Standard Normal Variate + 2nd order polynomial, 1st derivative, 11-point window Savitzky-Golay (SNV + SG1), 2nd order polynomial, 2nd derivative, 11-point window Savitzky-Golay (SG2), 2nd order polynomial, 2nd derivative, 11-point window Savitzky-Golay + Standard Normal Variate (SG2 + SNV), and Standard Normal Variate + 2nd order polynomial, 2nd derivative, 11-point window Savitzky-Golay (SNV + SG2).

As variable selection method we tested GA employing PLS, RF and kNN to compute the fitness function (see Table S5). To evaluate the quality of candidate solutions (i.e., collection of variables): for PLS and kNN, a 10-fold cross-validation approach was used to compute the Root Mean Square Error of Cross-Validation ($RMSE_{CV}$); while for RF, the Out-Of-Bag approach (OOB), typically computed with tree-based models, was used to compute the Root Mean Squared Error ($RMSE_{OOB}$). In particular, the normalized values, i.e.

$NRMSE_{CV}$ and $NRMSE_{OOB}$, computed by dividing $RMSE_{CV}$ and $RMSE_{OOB}$, respectively, by the maximum and minimum reference values in the property of interest (2000 and 1851, respectively), are used to calculate the fitness value.

Table S5. Summary of the parameter's settings of the variable selection methods implemented, including their abbreviations. For the genetic algorithm (GA): $size_{pop}$ is the population size, p_{cross} is the probability of crossover between pairs of individuals, p_{mut} is the probability of mutation in a parent individual, $iter_{max}$ is the maximum number of iterations to run before the GA search is halted. To calculate the fitness value for PLS-GA and kNN-GA, the normalized values of $RMSE_{CV}$, i.e. $NRMSE_{CV}$, was computed; while for RF-GA the normalized value of $RMSE_{OOB}$, i.e. $NRMSE_{OOB}$, was used. m_{try} is the number of attributes tried at each split, and n_{trees} is the number of trees in the forest.

Abbreviation	Variable selection method and parameters settings
PLS – GA	Genetic algorithm ($size_{pop} = 50$, $p_{cross} = 0.8$, $p_{mut} = 0.1$, $iter_{max} = 1000$) with $NRMSE_{CV}$ of PLS using 100 PLS components to calculate the fitness value
RF – GA	Genetic algorithm ($size_{pop} = 50$, $p_{cross} = 0.8$, $p_{mut} = 0.1$, $iter_{max} = 1000$) and $NRMSE_{OOB}$ of RF using $m_{try} = \sqrt{p}$ and $n_{trees} = 500$ to calculate the fitness value
kNN – GA	Genetic algorithm ($size_{pop} = 50$, $p_{cross} = 0.8$, $p_{mut} = 0.1$, $iter_{max} = 1000$) and $NRMSE_{CV}$ of kNN with previously tuned k values to calculate the fitness value. Euclidean distance. Kernel optimal.
Boruta	Variable selection using random forest using $m_{try} = p/3$ and $n_{trees} = 500$

Preliminary analyses (Figure S5-S7 and Tables S6-S8) were carried out using all variables (wavelengths). As result, for computation of fitness values in GA-based selection methods, we considered: 100 PLS components and chose the error at the minimum to calculate the fitness function using PLS (Table S6 and Figure S5); the number of neighbors (k) corresponding to the minimum value of error for each spectral preprocessing algorithm (Table S7 and Figure S6) to estimate the fitness function using kNN; 500 trees were chosen to estimate the fitness function using RF. At each node of the trees, a given number of randomly selected input variables (m_{try}) are chosen for all the trees in the forest. In general, m_{try} can be chosen as some function of the number of variable (p), usually $\sqrt{p}$ and p/3 are the default values for classification and regression, respectively. However, performance of RF is affected very little over a wide range of m_{try} values, except near the extremes (i.e., $m_{try} = 1$ or p)[15]. Based on preliminary tests (Figure S7), due to computational times of the GA implementation, we considered a random subsample of $\sqrt{p}$ of all available predictors (selected wavelengths) to determine the best split.

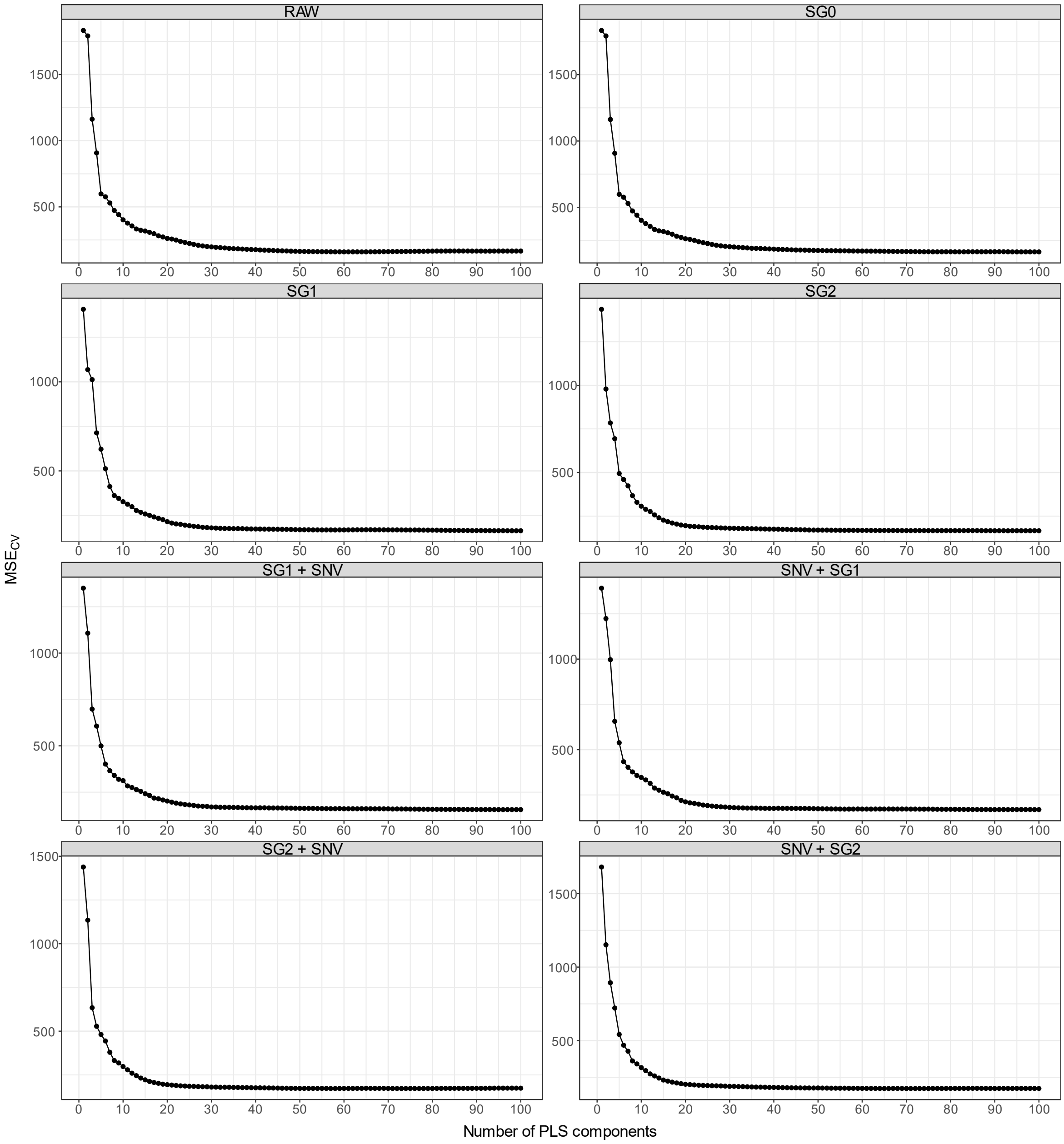

Figure S5. MSE_{CV} as a function of number of components for PLS. The minimum MSE_{CV} is reached before 100 components for all spectral preprocessing algorithms, as reported in Table S6.

Table S6. MSE_{CV} at the global minimum over 100 components for each spectral preprocessing algorithm using all variables (wavelengths).

Spectral preprocessing	Number of PLS components	MSE_{CV}
RAW	64	160.02
SG0	99	164.35
SG1	99	163.84
SG1+SNV	96	155.75
SNV+SG1	99	168.74
SG2	93	165.89
SG2+SNV	72	172.13
SNV+SG2	72	173.71

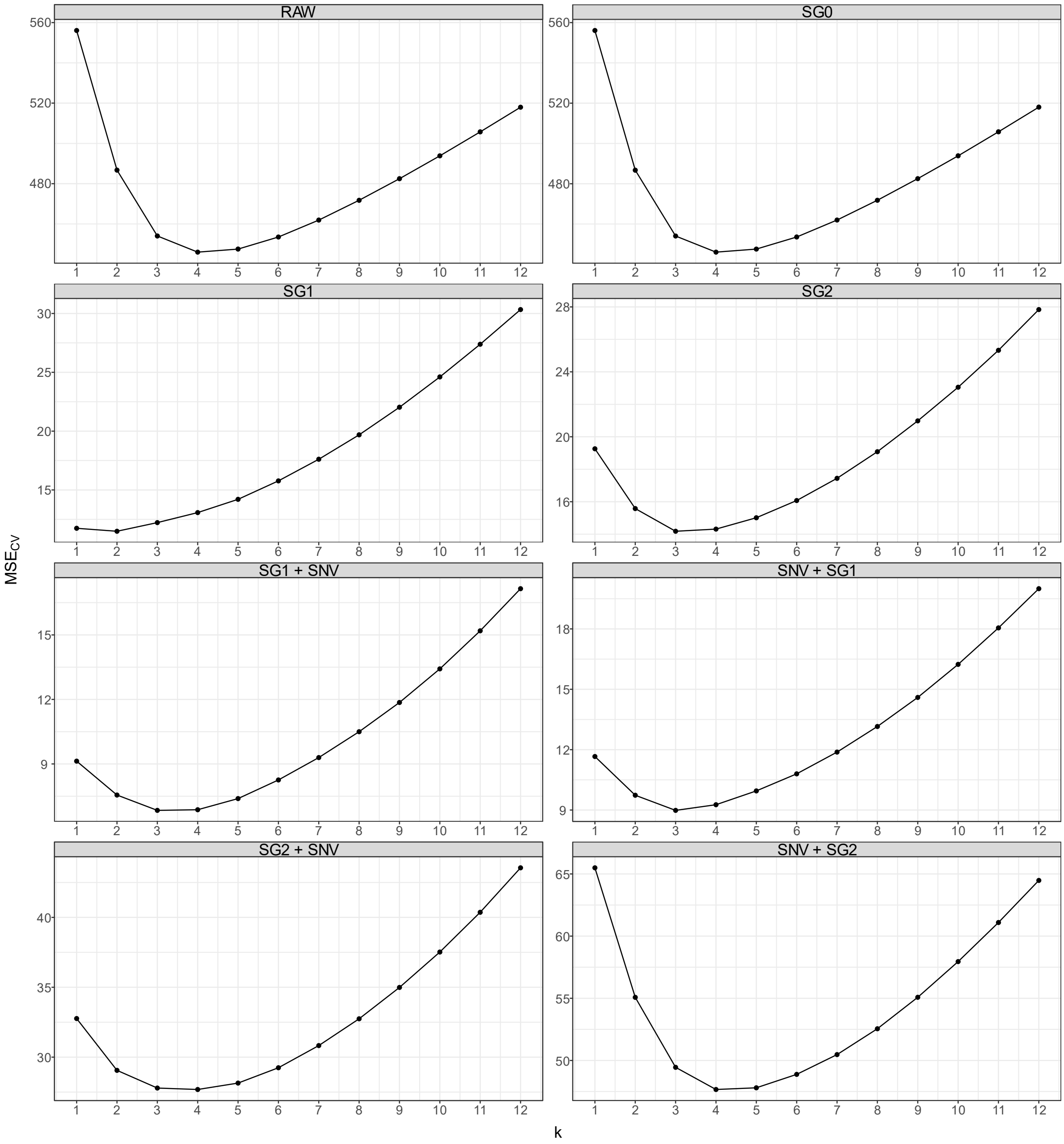

Figure S6. MSE_{CV} as a function of k values. The minimum MSE_{CV} corresponds to values of k ranging from 2 to 4, as reported in Table S7.

Table S7. MSE_{CV} at the minimum k value (from 1 to 12) for each spectral preprocessing algorithm using all variables (wavelengths).

Spectral preprocessing	k value	MSE_{CV}
RAW	4	446.01
SG0	4	446.06
SG1	2	11.49
SG1+SNV	3	6.84
SNV+SG1	3	8.98
SG2	3	14.31
SG2+SNV	4	27.68
SNV+SG2	4	47.66

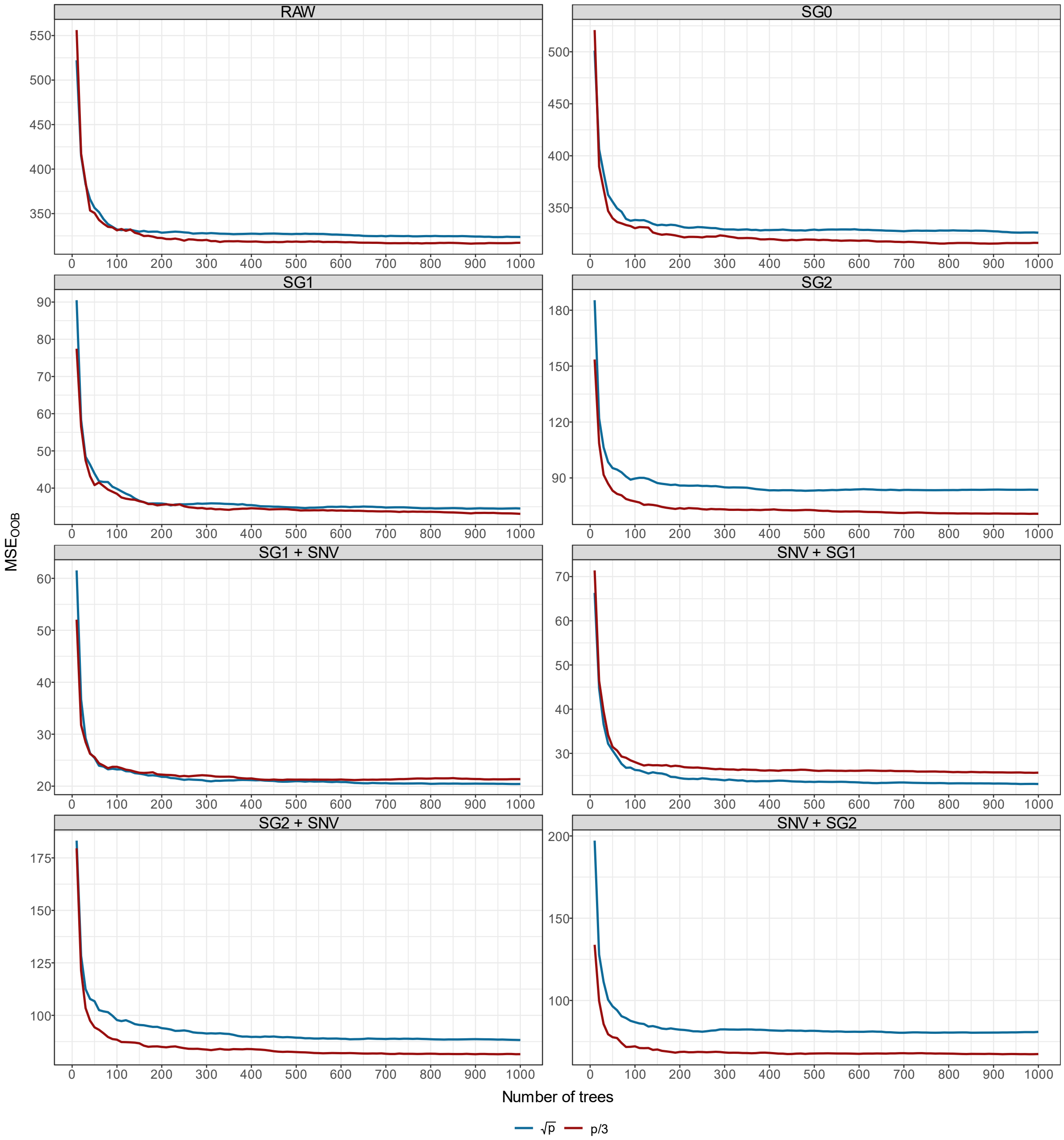

Figure S7. MSE_{OOB} sufficiently converge to a constant level after 400 trees for all spectral preprocessing algorithms.

The results of GA-based wavelength selection are shown in Figure S8-S10, demonstrating that a smooth convergence to a maximum fitness values of selected variables (wavelengths) is reached.

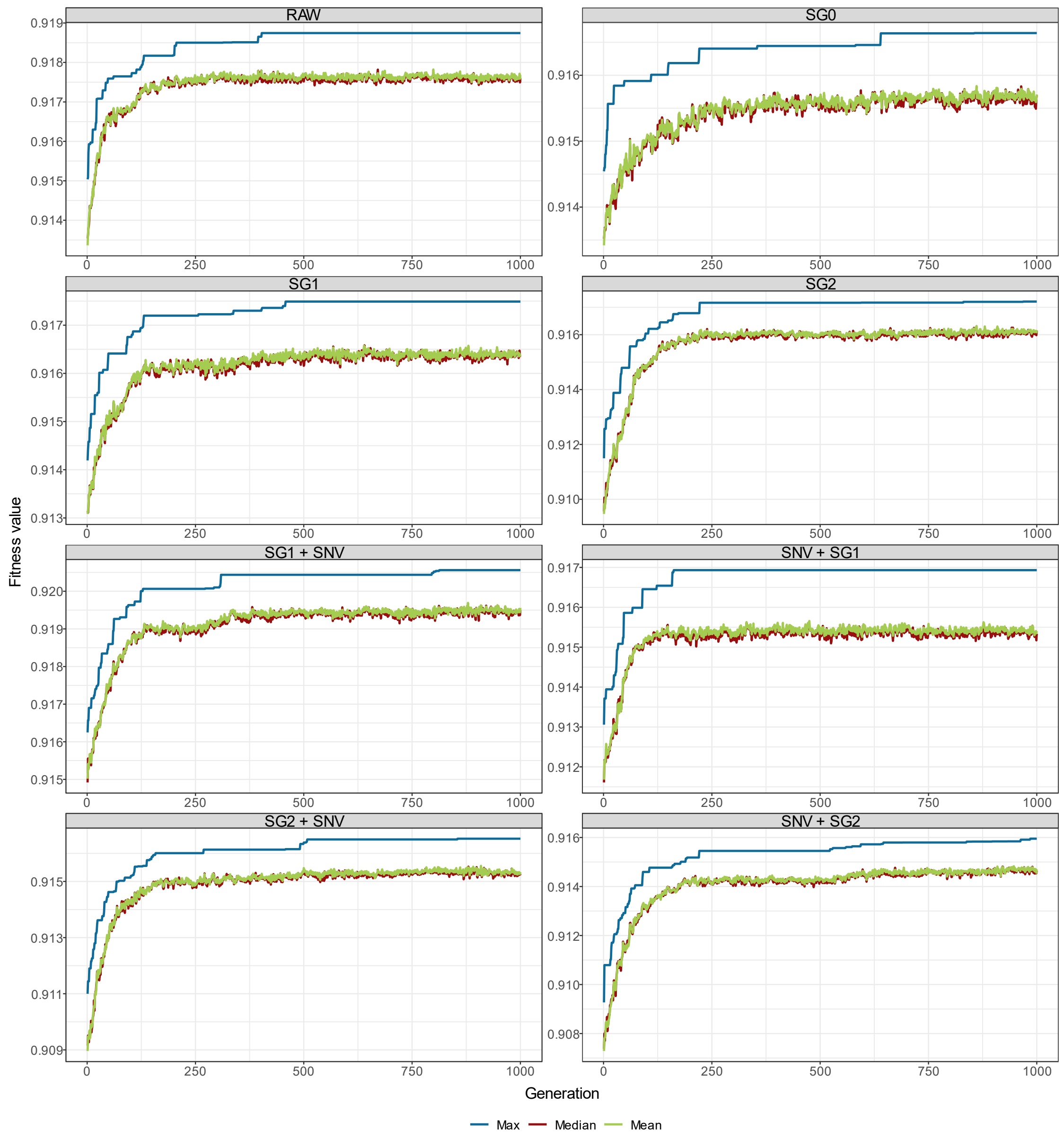

Figure S8. Fitness values for the PLS-GA run.

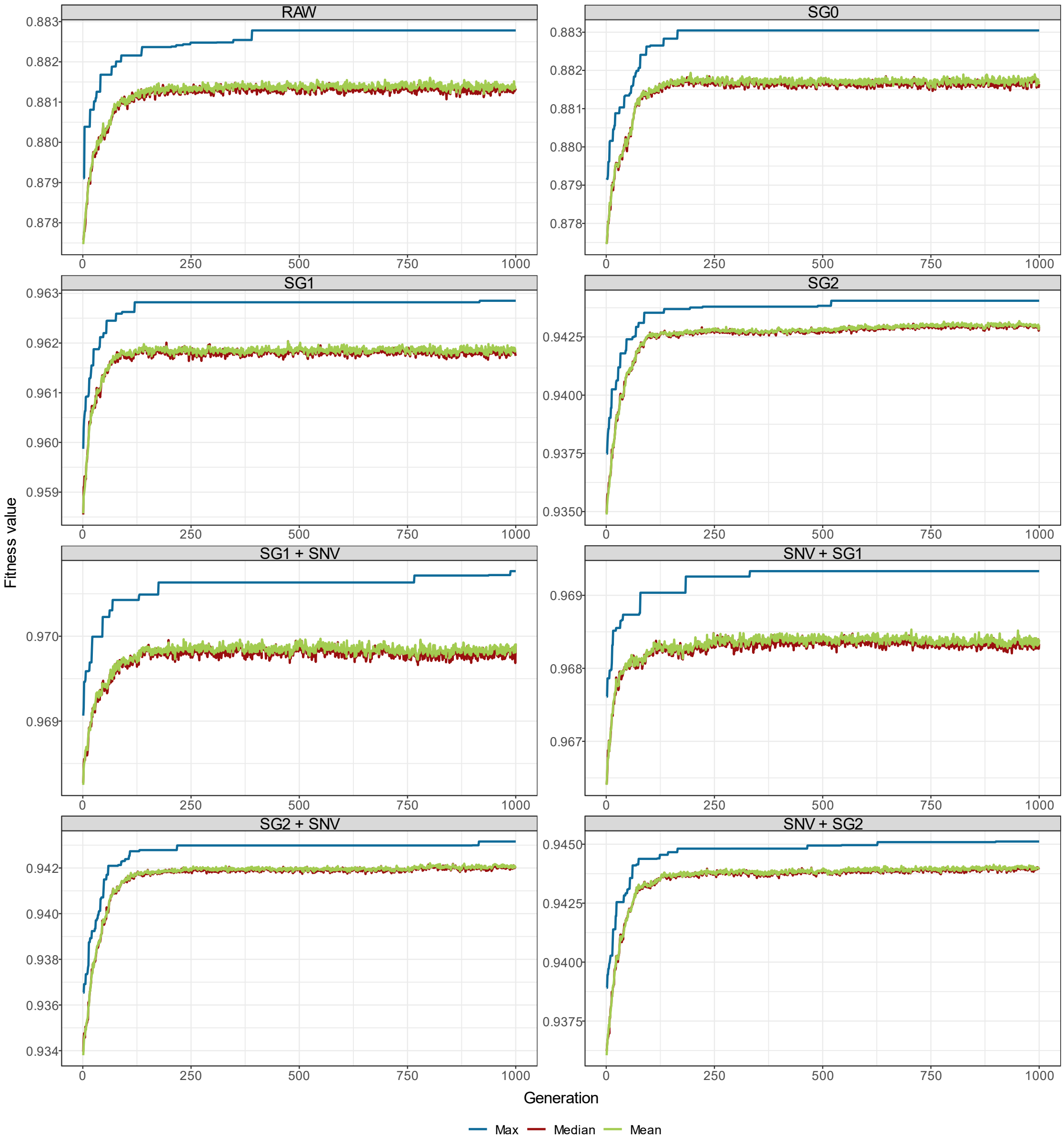

Figure S9. Fitness values for the RF-GA run.

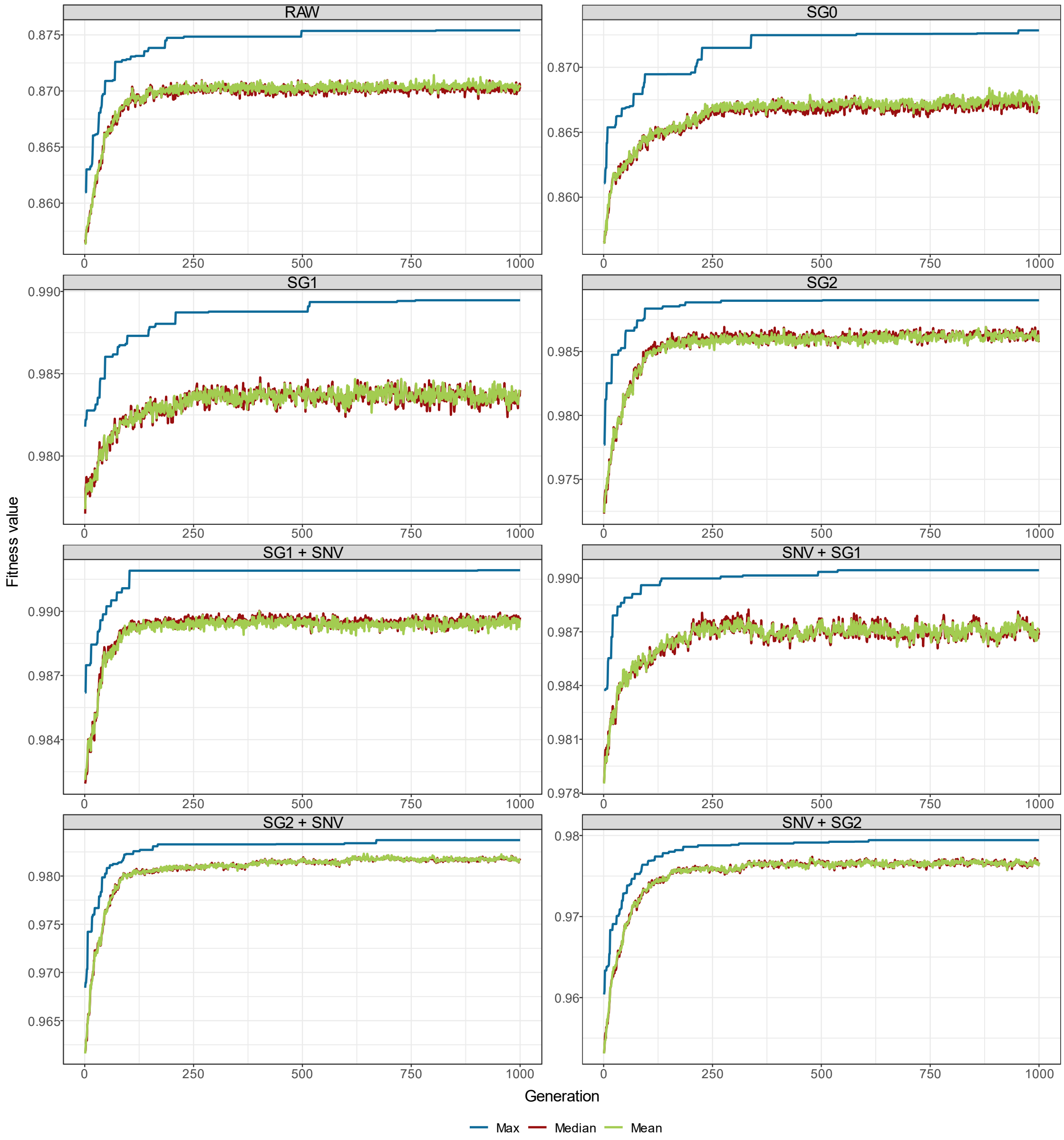

Figure S10. Fitness values for the kNN-GA run.

S2.3.2 Simulation Study

To investigate the trend of model performance according to sample size (i.e., number of spectra), we design a simulation study where 100 random samples for each of the ten sizes (i.e. 50, 100, 200, 300, 400, 500, 600, 700, 800, 900) are generated for the groups identified, by sampling without replacement from the original data. We stop at 900 as it is the maximum sample size of the smallest subset (i.e., "Front" and "Back" pages).

S3 Supplementary Figure

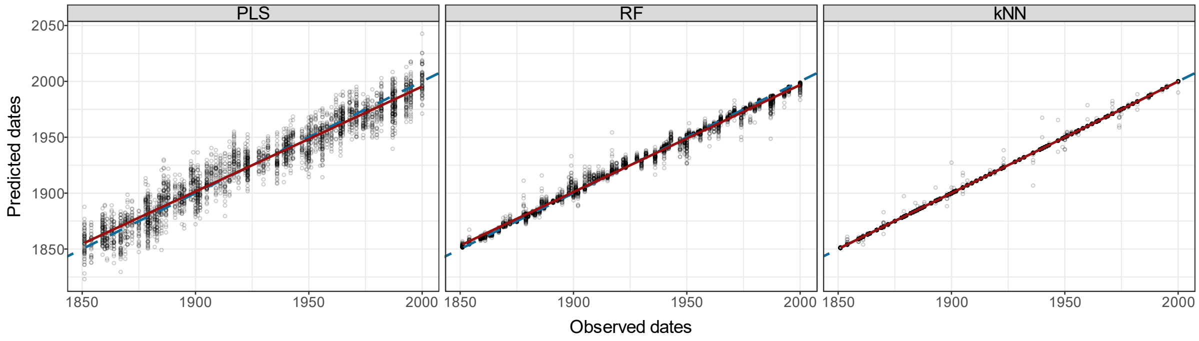

Figure S11. Predicted vs observed dates for PLS, RF and kNN built using the variables selected by PLS-GA, RF-GA and kNN-GA, respectively, and preprocessed by SG1 + SNV. The regression lines (red) and 1:1 lines (dashed blue) are also plotted.

S4 Supplementary Tables

Table S8. Summary of the results of the PLS models with different spectral preprocessing and variable selection methods in terms of $RMSE_{CV100}$, corresponding SD and CI95%.

PLS

	No Variable selection			Boruta			PLS – GA			RF – GA			kNN – GA		
	$RMSE_{CV100}$	SD	CI95%	$RMSE_{CV100}$	SD	CI95%	$RMSE_{CV100}$	SD	CI95%	$RMSE_{CV100}$	SD	CI95%	$RMSE_{CV100}$	SD	CI95%
**RAW**	12.62	0.05	[12.51,12.72]	14.27	0.05	[14.19,14.36]	12.27	0.05	[12.2,12.35]	12.98	0.05	[12.89,13.07]	13.15	0.05	[13.05,13.25]
**SG0**	12.78	0.05	[12.69,12.9]	14.19	0.05	[14.1,14.28]	12.58	0.05	[12.49,12.67]	12.87	0.05	[12.77,12.97]	12.93	0.05	[12.85,13.03]
**SG1**	12.78	0.05	[12.69,12.89]	12.82	0.05	[12.72,12.93]	12.46	0.05	[12.37,12.55]	12.92	0.05	[12.84,13.03]	13.08	0.04	[13,13.16]
**SG1 + SNV**	12.46	0.05	[12.36,12.55]	12.40	0.05	[12.31,12.5]	12.00	0.04	[11.92,12.08]	12.73	0.05	[12.63,12.82]	12.56	0.05	[12.46,12.65]
**SNV + SG1**	12.98	0.06	[12.88,13.1]	12.97	0.06	[12.85,13.07]	12.59	0.05	[12.5,12.7]	13.10	0.06	[12.98,13.2]	13.08	0.05	[12.99,13.18]
**SG2**	12.84	0.07	[12.71,12.97]	13.41	0.05	[13.33,13.52]	12.50	0.06	[12.38,12.62]	13.36	0.06	[13.24,13.48]	13.15	0.06	[13.04,13.29]
**SG2 + SNV**	13.02	0.06	[12.91,13.12]	13.80	0.04	[13.71,13.88]	12.61	0.05	[12.49,12.7]	13.69	0.05	[13.59,13.79]	13.57	0.05	[13.45,13.65]
**SNV + SG2**	13.16	0.07	[13.04,13.28]	13.90	0.06	[13.8,14.01]	12.70	0.06	[12.59,12.83]	13.90	0.06	[13.79,14]	13.79	0.06	[13.67,13.91]

Table S9. Summary of the results of the RF models with different spectral preprocessing and variable selection methods in terms of $RMSE_{CV100}$, corresponding SD and CI95%.

RF

	No Variable selection			Boruta			PLS – GA			RF – GA			kNN – GA		
	$RMSE_{CV100}$	SD	CI95%	$RMSE_{CV100}$	SD	CI95%	$RMSE_{CV100}$	SD	CI95%	$RMSE_{CV100}$	SD	CI95%	$RMSE_{CV100}$	SD	CI95%
**RAW**	18.47	0.11	[18.25,18.69]	18.05	0.11	[17.85,18.26]	18.69	0.11	[18.47,18.86]	18.13	0.10	[17.93,18.31]	18.45	0.11	[18.22,18.63]
**SG0**	18.46	0.11	[18.26,18.65]	18.04	0.11	[17.81,18.23]	18.72	0.10	[18.52,18.92]	18.06	0.10	[17.84,18.23]	18.55	0.11	[18.33,18.74]
**SG1**	6.18	0.05	[6.1,6.29]	6.08	0.04	[6.02,6.17]	6.44	0.04	[6.37,6.52]	5.90	0.04	[5.83,5.98]	6.25	0.04	[6.18,6.34]
**SG1 + SNV**	4.77	0.04	[4.69,4.84]	4.68	0.04	[4.61,4.77]	4.87	0.04	[4.8,4.94]	4.68	0.04	[4.62,4.76]	4.84	0.04	[4.77,4.92]
**SNV + SG1**	5.04	0.04	[4.96,5.11]	4.92	0.04	[4.85,4.99]	5.22	0.04	[5.15,5.28]	4.90	0.03	[4.84,4.96]	5.09	0.04	[5.02,5.15]
**SG2**	9.42	0.04	[9.35,9.49]	8.64	0.04	[8.57,8.71]	9.60	0.04	[9.53,9.67]	8.74	0.03	[8.67,8.83]	9.72	0.04	[9.64,9.8]
**SG2 + SNV**	9.71	0.04	[9.64,9.79]	8.95	0.04	[8.87,9.04]	9.92	0.04	[9.84,9.99]	8.90	0.04	[8.83,8.97]	9.61	0.04	[9.54,9.69]
**SNV + SG2**	9.28	0.03	[9.2,9.34]	8.48	0.04	[8.41,8.54]	9.53	0.04	[9.45,9.59]	8.59	0.04	[8.53,8.66]	9.33	0.04	[9.26,9.41]

Table S10. Summary of the results of the kNN models with different spectral preprocessing and variable selection methods in in terms of $RMSE_{CV100}$, corresponding SD and CI95%.

	kNN														
	No Variable selection			Boruta			PLS – GA			RF – GA			kNN – GA		
	$RMSE_{CV100}$	SD	CI95%	$RMSE_{CV100}$	SD	CI95%	$RMSE_{CV100}$	SD	CI95%	$RMSE_{CV100}$	SD	CI95%	$RMSE_{CV100}$	SD	CI95%
**RAW**	21.33	0.26	[20.92,21.85]	18.41	0.22	[18.05,18.9]	21.30	0.26	[20.88,21.8]	20.97	0.25	[20.58,21.5]	19.40	0.24	[19.02,19.94]
**SG0**	21.33	0.26	[20.92,21.85]	18.40	0.22	[18.05,18.9]	21.79	0.26	[21.36,22.32]	20.88	0.26	[20.47,21.44]	19.88	0.24	[19.53,20.39]
**SG1**	3.28	0.28	[2.88,3.81]	2.94	0.26	[2.6,3.45]	3.80	0.30	[3.37,4.44]	3.75	0.26	[3.39,4.24]	2.53	0.32	[2.08,3.07]
**SG1 + SNV**	2.77	0.18	[2.47,3.08]	2.46	0.21	[2.13,2.89]	2.63	0.19	[2.36,3]	2.74	0.18	[2.44,3.1]	1.57	0.13	[1.36,1.86]
**SNV + SG1**	3.24	0.20	[2.84,3.66]	2.94	0.21	[2.59,3.41]	3.04	0.21	[2.67,3.52]	2.94	0.23	[2.49,3.43]	2.09	0.21	[1.6,2.47]
**SG2**	3.63	0.21	[3.2,4]	3.65	0.23	[3.31,4.16]	3.77	0.24	[3.36,4.22]	3.53	0.24	[3.09,3.97]	2.12	0.21	[1.75,2.54]
**SG2 + SNV**	5.37	0.14	[5.11,5.67]	3.58	0.16	[3.31,3.91]	4.39	0.15	[4.12,4.73]	4.12	0.16	[3.86,4.44]	2.75	0.13	[2.56,3.05]
**SNV + SG2**	6.82	0.15	[6.54,7.13]	5.21	0.16	[4.89,5.48]	5.64	0.15	[5.38,5.92]	5.06	0.15	[4.82,5.38]	3.50	0.16	[3.24,3.82]

Table S11. Number of selected variables (wavelengths) for each combination of spectral preprocessing and variable selection method. The percentages of the initial number of variables are reported in brackets.

	Boruta	PLS – GA	RF – GA	kNN – GA
**RAW**	658 (55%)	608 (51%)	606 (51%)	542 (45%)
**SG0**	659 (55%)	578 (48%)	578 (48%)	555 (46%)
**SG1**	1021 (85%)	580 (48%)	587 (49%)	591 (49%)
**SG1 + SNV**	1036 (86%)	599 (50%)	607 (51%)	614 (51%)
**SNV + SG1**	1003 (84%)	593 (49%)	585 (49%)	608 (51%)
**SG2**	680 (57%)	614 (51%)	583 (49%)	590 (49%)
**SG2 + SNV**	666 (56%)	587 (49%)	546 (46%)	554 (46%)
**SNV + SG2**	652 (54%)	612 (51%)	578 (48%)	585 (49%)

Table S12. Summary of the results of t-test (two-tails) for the two best models specified with PLS, RF, and kNN.

SML method	Spectral preprocessing	Variable selection	Mean (y)	n	Differences		
					t	df	p-value
PLS	SG1 + SNV	PLS – GA	11.997	100	43.35	197.81	< 2.2e-16
	RAW	PLS – GA	12.271	100			
RF	SG1 + SNV	Boruta	4.678	100	-0.69	194.82	0.4908
	SG1 + SNV	RF – GA	4.682	100			
kNN	SG1 + SNV	kNN – GA	1.574	100	-20.95	169.86	< 2.2e-16
	SNV + SG1	kNN – GA	2.089	100			

Table S13. Correlation matrix of the variable importance between the common variables of the three SML methods.

	PLS	RF	kNN
**PLS**	1.000	-	-
**RF**	0.956	1.000	-
**kNN**	0.951	0.999	1.000

Table S14. Summary of the results in terms of $RMSE_{CV100}$, corresponding SD and CI95% of PLS built using different number of spectra grouped by the publication date of the books (1851-1900, 1901-1950 and 1951-2000). The $NRMSE_{CV100}$ is also reported.

	PLS											
	Publication date											
	1851-1900				1901-1950				1951-2000			
Number of spectra	$NRMSE_{CV100}$	$RMSE_{CV100}$	SD	CI95%	$NRMSE_{CV100}$	$RMSE_{CV100}$	SD	CI95%	$NRMSE_{CV100}$	$RMSE_{CV100}$	SD	CI95%
50	0.12	5.65	0.82	[4.24, 7.21]	0.25	12.32	1.63	[8.74, 14.81]	0.16	7.65	1.36	[5.12, 10.24]
100	0.10	4.90	0.40	[4.15, 5.68]	0.21	10.09	1.03	[8.16, 12.05]	0.12	6.00	0.65	[4.82, 7.29]
200	0.09	4.38	0.28	[3.95, 5.00]	0.17	8.45	0.58	[7.29, 9.39]	0.10	4.97	0.36	[4.30, 5.53]
300	0.08	4.12	0.20	[3.79, 4.51]	0.15	7.55	0.41	[6.89, 8.43]	0.09	4.57	0.28	[4.13, 5.27]
400	0.08	3.89	0.17	[3.62, 4.23]	0.14	7.02	0.31	[6.41, 7.55]	0.09	4.29	0.25	[3.91, 4.82]
500	0.08	3.74	0.11	[3.52, 3.97]	0.14	6.73	0.24	[6.31, 7.23]	0.08	4.14	0.19	[3.82, 4.49]
600	0.07	3.62	0.10	[3.46, 3.79]	0.13	6.52	0.19	[6.10, 6.86]	0.08	3.98	0.15	[3.69, 4.23]
700	0.07	3.52	0.08	[3.34, 3.68]	0.13	6.35	0.16	[6.06, 6.64]	0.08	3.85	0.16	[3.58, 4.09]
800	0.07	3.44	0.06	[3.33, 3.54]	0.13	6.23	0.10	[6.05, 6.40]	0.08	3.80	0.11	[3.56, 3.96]
900	0.07	3.38	0.05	[3.31, 3.47]	0.13	6.15	0.07	[6.00, 6.29]	0.08	3.73	0.09	[3.52, 3.86]

Table S15. Summary of the results in terms of $RMSE_{CV100}$, corresponding SD and CI95% of the RF models built using different number of spectra grouped by the publication date of the books (1851-1900, 1901-1950 and 1951-2000). The $NRMSE_{CV100}$ is also reported.

RF												
	Publication date											
	1851-1900				1901-1950				1951-2000			
Number of spectra	$NRMSE_{CV100}$	$RMSE_{CV100}$	SD	CI95%	$NRMSE_{CV100}$	$RMSE_{CV100}$	SD	CI95%	$NRMSE_{CV100}$	$RMSE_{CV100}$	SD	CI95%
50	0.13	6.30	0.70	[4.88, 7.57]	0.25	12.35	1.09	[10.25, 14.34]	0.18	8.91	0.82	[7.11, 10.13]
100	0.11	5.22	0.35	[4.59, 5.84]	0.22	10.59	0.69	[9.22, 12.04]	0.15	7.25	0.43	[6.26, 7.96]
200	0.08	4.08	0.18	[3.74, 4.41]	0.17	8.56	0.45	[7.70, 9.47]	0.11	5.59	0.22	[5.20, 5.95]
300	0.07	3.40	0.15	[3.15, 3.67]	0.15	7.27	0.28	[6.69, 7.75]	0.09	4.65	0.15	[4.40, 4.93]
400	0.06	2.95	0.10	[2.74, 3.12]	0.13	6.41	0.23	[5.95, 6.78]	0.08	4.07	0.12	[3.83, 4.28]
500	0.05	2.63	0.08	[2.48, 2.77]	0.12	5.81	0.18	[5.48, 6.14]	0.07	3.66	0.10	[3.51, 3.85]
600	0.05	2.38	0.06	[2.27, 2.49]	0.11	5.35	0.13	[5.08, 5.56]	0.07	3.34	0.07	[3.22, 3.48]
700	0.04	2.17	0.06	[2.06, 2.27]	0.10	4.99	0.10	[4.80, 5.16]	0.06	3.10	0.06	[2.98, 3.20]
800	0.04	2.02	0.04	[1.94, 2.10]	0.10	4.69	0.08	[4.56, 4.86]	0.06	2.90	0.05	[2.78, 3.00]
900	0.04	1.89	0.03	[1.84, 1.96]	0.09	4.44	0.06	[4.33, 4.54]	0.06	2.73	0.04	[2.65, 2.80]

Table S16. Summary of the results in terms of $RMSE_{CV100}$, corresponding SD and CI95% of the kNN models built using different number of spectra grouped by the publication date of the books (1851-1900, 1901-1950 and 1951-2000). The $NRMSE_{CV100}$ is also reported.

kNN												
	Publication date											
	1851-1900				1901-1950				1951-2000			
Number of spectra	$NRMSE_{CV100}$	$RMSE_{CV100}$	SD	CI95%	$NRMSE_{CV100}$	$RMSE_{CV100}$	SD	CI95%	$NRMSE_{CV100}$	$RMSE_{CV100}$	SD	CI95%
50	0.15	7.22	0.91	[5.40, 8.80]	0.25	12.31	2.03	[9.09, 16.44]	0.18	8.85	1.10	[6.91, 10.71]
100	0.11	5.39	0.59	[4.31, 6.67]	0.19	9.38	1.14	[6.92, 11.61]	0.13	6.46	0.98	[4.53, 8.10]
200	0.07	3.40	0.42	[2.65, 4.16]	0.12	5.99	0.89	[4.30, 7.68]	0.08	4.09	0.58	[3.11, 5.05]
300	0.05	2.39	0.31	[1.88, 3.07]	0.09	4.21	0.65	[3.03, 5.45]	0.06	2.84	0.40	[2.04, 3.58]
400	0.04	1.86	0.26	[1.39, 2.30]	0.07	3.25	0.59	[2.16, 4.39]	0.04	2.09	0.30	[1.55, 2.69]
500	0.03	1.49	0.21	[1.13, 1.90]	0.05	2.51	0.46	[1.72, 3.40]	0.03	1.63	0.28	[1.08, 2.13]
600	0.03	1.25	0.17	[0.93, 1.53]	0.05	2.21	0.42	[1.64, 3.13]	0.03	1.38	0.23	[0.97, 1.88]
700	0.02	1.08	0.14	[0.84, 1.32]	0.04	1.81	0.34	[1.20, 2.48]	0.02	1.19	0.18	[0.78, 1.49]
800	0.02	0.95	0.11	[0.76, 1.18]	0.03	1.58	0.26	[1.09, 2.08]	0.02	1.01	0.15	[0.75, 1.27]
900	0.02	0.86	0.09	[0.67, 1.02]	0.03	1.38	0.20	[1.05, 1.80]	0.02	0.95	0.13	[0.72, 1.14]

Table S17. Summary of the results in terms of $RMSE_{CV100}$, corresponding SD and CI95% of PLS, RF, and kNN built using different number of spectra without grouping. The $NRMSE_{CV100}$ is also reported.

	All spectra – No subsets											
	PLS				RF				kNN			
Number of spectra	$NRMSE_{CV100}$	$RMSE_{CV100}$	SD	CI95%	$NRMSE_{CV100}$	$RMSE_{CV100}$	SD	CI95%	$NRMSE_{CV100}$	$RMSE_{CV100}$	SD	CI95%
50	0.15	21.70	3.05	[16.65, 27.15]	0.14	21.07	2.04	[16.64, 25.07]	0.21	31.37	3.94	[24.49, 38.83]
100	0.13	19.86	2.09	[15.91, 24.03]	0.12	17.75	1.10	[15.17, 19.71]	0.17	25.21	2.84	[20.12, 30.79]
200	0.12	18.12	1.11	[16.12, 20.26]	0.10	14.89	0.75	[13.46, 16.31]	0.13	19.65	1.85	[15.99, 23.17]
300	0.12	17.23	0.84	[15.86, 18.61]	0.09	13.25	0.58	[12.17, 14.32]	0.11	16.15	1.35	[13.77, 18.88]
400	0.11	16.41	0.67	[15.24, 17.69]	0.08	11.95	0.42	[11.09, 12.60]	0.09	13.17	1.39	[10.60, 15.74]
500	0.11	15.82	0.54	[14.70, 16.95]	0.07	11.00	0.36	[10.40, 11.71]	0.08	11.30	1.19	[9.16, 13.64]
600	0.10	15.28	0.53	[14.40, 16.34]	0.07	10.19	0.28	[9.65, 10.68]	0.06	9.54	0.92	[7.73, 11.26]
700	0.10	14.89	0.44	[14.01, 15.69]	0.06	9.55	0.25	[9.06, 10.01]	0.06	8.52	0.87	[6.78, 10.07]
800	0.10	14.64	0.37	[13.94, 15.27]	0.06	8.98	0.22	[8.58, 9.42]	0.05	7.33	0.76	[5.89, 8.97]
900	0.10	14.42	0.33	[13.91, 15.08]	0.06	8.52	0.21	[8.09, 8.92]	0.04	6.46	0.76	[5.13, 8.03]

Table S18. Summary of the results in terms of $RMSE_{CV100}$, corresponding SD and CI95% of the PLS models built using different number of spectra grouped by the page (front, middle, back pages of the book block) where the measurement was made. The $NRMSE_{CV100}$ is also reported.

	PLS											
	Page											
	Front pages				Middle pages				Back pages			
Number of spectra	$NRMSE_{CV100}$	$RMSE_{CV100}$	SD	CI95%	$NRMSE_{CV100}$	$RMSE_{CV100}$	SD	CI95%	$NRMSE_{CV100}$	$RMSE_{CV100}$	SD	CI95%
50	0.15	21.85	2.76	[16.02, 27.76]	0.15	21.65	3.05	[16.11, 27.06]	0.14	21.47	2.74	[16.36, 27.44]
100	0.14	20.37	1.79	[17.52, 23.92]	0.13	19.46	1.66	[16.22, 22.59]	0.13	20.02	1.73	[16.87, 22.90]
200	0.13	18.77	1.06	[16.54, 20.63]	0.12	17.81	1.07	[15.83, 20.18]	0.12	18.33	1.05	[16.24, 20.14]
300	0.12	17.63	0.82	[16.31, 19.15]	0.11	16.77	0.84	[15.05, 18.23]	0.11	17.09	0.71	[15.82, 18.42]
400	0.11	16.95	0.61	[15.63, 17.99]	0.11	15.97	0.64	[14.89, 17.11]	0.11	16.28	0.60	[15.30, 17.27]
500	0.11	16.26	0.44	[15.53, 17.07]	0.10	15.31	0.48	[14.31, 16.24]	0.10	15.59	0.54	[14.56, 16.54]
600	0.11	15.83	0.35	[15.16, 16.45]	0.10	14.95	0.40	[14.30, 15.61]	0.10	15.07	0.37	[14.24, 15.74]
700	0.10	15.42	0.27	[14.92, 15.93]	0.10	14.53	0.36	[13.83, 15.24]	0.10	14.69	0.25	[14.24, 15.20]
800	0.10	15.12	0.19	[14.77, 15.47]	0.10	14.35	0.26	[13.84, 14.82]	0.10	14.43	0.20	[14.04, 14.76]
900	0.10	14.93	0.12	[14.72, 15.20]	0.10	14.16	0.23	[13.71, 14.60]	0.10	14.21	0.11	[13.98, 14.44]

Table S19. Summary of the results in terms of $RMSE_{CV100}$, corresponding SD and CI95% of the RF models built using different number of spectra grouped by the page (front, middle, back pages of the book block) where the measurement was made. The $NRMSE_{CV100}$ is also reported.

	RF											
	Page											
	Front pages				Middle pages				Back pages			
Number of spectra	$NRMSE_{CV100}$	$RMSE_{CV100}$	SD	CI95%	$NRMSE_{CV100}$	$RMSE_{CV100}$	SD	CI95%	$NRMSE_{CV100}$	$RMSE_{CV100}$	SD	CI95%
50	0.14	20.37	1.86	[16.37, 23.98]	0.14	20.68	1.71	[17.57, 23.99]	0.14	20.37	2.07	[16.16, 23.64]
100	0.12	17.98	1.12	[16.03, 20.42]	0.12	17.81	1.23	[15.00, 20.17]	0.12	17.86	1.27	[15.36, 20.24]
200	0.10	15.04	0.68	[13.60, 16.32]	0.10	14.97	0.64	[13.91, 16.28]	0.10	14.82	0.71	[13.20, 16.31]
300	0.09	13.29	0.48	[12.42, 14.09]	0.09	13.14	0.51	[12.20, 14.16]	0.09	12.97	0.55	[11.91, 13.88]
400	0.08	11.95	0.41	[11.29, 12.71]	0.08	11.74	0.45	[10.94, 12.67]	0.08	11.62	0.38	[10.95, 12.35]
500	0.07	10.90	0.31	[10.39, 11.44]	0.07	10.78	0.31	[10.26, 11.44]	0.07	10.58	0.28	[9.97, 11.13]
600	0.07	10.06	0.21	[9.64, 10.46]	0.07	9.88	0.23	[9.37, 10.24]	0.07	9.69	0.19	[9.35, 10.04]
700	0.06	9.32	0.18	[9.03, 9.72]	0.06	9.14	0.20	[8.78, 9.50]	0.06	8.94	0.16	[8.62, 9.28]
800	0.06	8.72	0.14	[8.51, 8.98]	0.06	8.55	0.15	[8.21, 8.79]	0.06	8.33	0.13	[8.05, 8.59]
900	0.06	8.22	0.11	[8.05, 8.49]	0.05	8.03	0.16	[7.75, 8.33]	0.05	7.79	0.13	[7.58, 8.06]

Table S20. Summary of the results in terms of $RMSE_{CV100}$, corresponding SD and CI95% of the kNN models built using different number of spectra grouped by the page (front, middle, back pages of the book block) where the measurement was made. The $NRMSE_{CV100}$ is also reported.

	kNN											
	Page											
	Front pages				Middle pages				Back pages			
Number of spectra	$NRMSE_{CV100}$	$RMSE_{CV100}$	SD	CI95%	$NRMSE_{CV100}$	$RMSE_{CV100}$	SD	CI95%	$NRMSE_{CV100}$	$RMSE_{CV100}$	SD	CI95%
50	0.20	30.37	4.12	[23.28, 38.58]	0.21	30.71	3.79	[23.40, 37.92]	0.21	31.12	4.74	[22.14, 40.21]
100	0.17	25.93	2.60	[21.90, 31.83]	0.17	25.05	3.00	[19.76, 30.91]	0.18	26.73	2.87	[21.36, 31.89]
200	0.14	20.29	1.59	[17.34, 23.57]	0.13	19.23	1.66	[16.25, 22.74]	0.14	20.74	1.57	[18.22, 23.90]
300	0.11	16.08	1.41	[13.51, 18.71]	0.10	15.24	1.29	[13.20, 17.76]	0.11	16.51	1.60	[13.72, 19.33]
400	0.09	13.23	1.18	[11.05, 15.42]	0.08	12.31	1.05	[10.12, 14.18]	0.09	13.35	1.14	[11.17, 15.55]
500	0.07	10.78	0.98	[8.97, 12.60]	0.07	10.10	0.88	[8.54, 11.79]	0.07	10.86	0.92	[9.44, 12.48]
600	0.06	8.81	0.71	[7.47, 10.19]	0.06	8.27	0.78	[6.67, 9.72]	0.06	8.97	0.95	[7.17, 10.83]
700	0.05	7.19	0.65	[6.03, 8.50]	0.05	6.80	0.71	[5.61, 8.16]	0.05	7.49	0.78	[6.02, 9.12]
800	0.04	5.94	0.59	[4.81, 6.99]	0.04	5.57	0.68	[4.47, 6.86]	0.04	6.07	0.65	[4.97, 7.24]
900	0.03	5.07	0.33	[4.62, 5.82]	0.03	4.58	0.59	[3.63, 5.97]	0.03	4.79	0.56	[4.00, 5.98]

Table S21. Summary of the results in terms of $RMSE_{CV100}$, corresponding SD and CI95% of the PLS models built using different number of spectra grouped by the point (gutter, centre, margin of the page) where the measurement was made. The $NRMSE_{CV100}$ is also reported.

PLS												
	Point											
	Gutter				Center				Margin			
Number of spectra	$NRMSE_{CV100}$	$RMSE_{CV100}$	SD	CI95%	$NRMSE_{CV100}$	$RMSE_{CV100}$	SD	CI95%	$NRMSE_{CV100}$	$RMSE_{CV100}$	SD	CI95%
50	0.14	20.17	3.04	[14.35, 26.52]	0.13	19.68	2.71	[14.96, 25.06]	0.14	20.60	2.93	[15.65, 26.51]
100	0.12	18.34	1.66	[15.04, 21.04]	0.12	17.71	1.94	[14.07, 21.40]	0.13	18.88	1.75	[15.47, 22.18]
200	0.11	16.40	1.09	[14.65, 18.81]	0.11	16.19	0.95	[14.25, 18.07]	0.11	16.99	1.06	[14.85, 18.83]
300	0.10	15.27	0.81	[13.66, 16.91]	0.10	14.93	0.77	[13.71, 16.47]	0.11	15.78	0.76	[14.49, 17.33]
400	0.10	14.67	0.61	[13.45, 15.72]	0.10	14.16	0.53	[13.18, 15.13]	0.10	14.95	0.58	[13.94, 15.89]
500	0.09	14.06	0.55	[13.12, 15.12]	0.09	13.60	0.42	[12.83, 14.41]	0.10	14.32	0.50	[13.40, 15.27]
600	0.09	13.64	0.43	[12.80, 14.38]	0.09	13.12	0.37	[12.40, 13.84]	0.09	13.86	0.36	[13.20, 14.53]
700	0.09	13.36	0.33	[12.73, 14.01]	0.09	12.74	0.34	[12.09, 13.32]	0.09	13.54	0.27	[13.02, 13.96]
800	0.09	13.18	0.25	[12.67, 13.62]	0.08	12.53	0.21	[12.13, 12.90]	0.09	13.32	0.22	[12.90, 13.74]
900	0.09	12.98	0.19	[12.64, 13.30]	0.08	12.26	0.16	[11.97, 12.55]	0.09	13.10	0.16	[12.80, 13.37]

Table S22. Summary of the results in terms of $RMSE_{CV100}$, corresponding SD and CI95% of the RF models built using different number of spectra grouped by the point (gutter, centre, margin of the page) where the measurement was made. The $NRMSE_{CV100}$ is also reported.

RF												
	Point											
	Gutter				Center				Margin			
Number of spectra	$NRMSE_{CV100}$	$RMSE_{CV100}$	SD	CI95%	$NRMSE_{CV100}$	$RMSE_{CV100}$	SD	CI95%	$NRMSE_{CV100}$	$RMSE_{CV100}$	SD	CI95%
50	0.13	19.42	1.83	[15.96, 23.25]	0.13	19.00	1.94	[15.50, 23.05]	0.13	19.45	1.77	[15.83, 22.61]
100	0.11	16.14	1.02	[14.46, 18.08]	0.11	15.88	1.20	[13.74, 17.92]	0.11	16.22	1.08	[14.18, 18.27]
200	0.09	12.83	0.56	[11.80, 13.85]	0.09	12.86	0.58	[11.67, 13.91]	0.09	12.95	0.56	[11.82, 13.92]
300	0.07	10.73	0.45	[9.92, 11.53]	0.07	11.04	0.36	[10.34, 11.68]	0.07	10.78	0.43	[10.04, 11.63]
400	0.06	9.40	0.35	[8.66, 10.05]	0.07	9.78	0.29	[9.24, 10.35]	0.06	9.31	0.29	[8.75, 9.80]
500	0.06	8.39	0.29	[7.85, 8.90]	0.06	8.82	0.23	[8.38, 9.28]	0.05	8.17	0.24	[7.73, 8.65]
600	0.05	7.64	0.23	[7.21, 8.12]	0.05	8.10	0.19	[7.71, 8.42]	0.05	7.36	0.21	[7.02, 7.79]
700	0.05	7.06	0.18	[6.77, 7.39]	0.05	7.54	0.17	[7.19, 7.90]	0.04	6.66	0.15	[6.40, 6.95]
800	0.04	6.57	0.15	[6.25, 6.83]	0.05	7.07	0.14	[6.82, 7.34]	0.04	6.15	0.12	[5.88, 6.40]
900	0.04	6.19	0.10	[5.99, 6.37]	0.04	6.69	0.10	[6.54, 6.87]	0.04	5.70	0.09	[5.55, 5.87]

Table S23. Summary of the results in terms of $RMSE_{CV100}$, corresponding SD and CI95% of the kNN models built using different number of spectra grouped by the point (gutter, centre, margin of the page) where the measurement was made. The $NRMSE_{CV100}$ is also reported.

	kNN											
	Point											
	Gutter				Center				Margin			
**Number of spectra**	**$NRMSE_{CV100}$**	**$RMSE_{CV100}$**	**SD**	**CI95%**	**$NRMSE_{CV100}$**	**$RMSE_{CV100}$**	**SD**	**CI95%**	**$NRMSE_{CV100}$**	**$RMSE_{CV100}$**	**SD**	**CI95%**
50	0.17	25.46	3.86	[18.22, 31.79]	0.16	23.63	3.61	[16.45, 30.20]	0.17	25.48	3.69	[19.66, 32.03]
100	0.13	19.78	2.43	[14.66, 24.24]	0.13	19.02	2.37	[14.59, 23.14]	0.13	19.43	2.57	[14.93, 24.65]
200	0.09	13.30	1.64	[10.10, 16.58]	0.09	13.24	1.46	[10.79, 15.98]	0.09	12.70	1.58	[10.05, 15.96]
300	0.06	8.82	1.19	[6.56, 11.52]	0.06	9.35	1.24	[6.64, 11.69]	0.06	8.41	1.03	[6.66, 10.83]
400	0.04	6.05	1.12	[4.16, 8.51]	0.05	6.73	1.01	[4.94, 8.64]	0.04	5.83	0.98	[4.11, 7.75]
500	0.03	4.22	0.74	[2.80, 5.72]	0.03	5.10	0.74	[3.63, 6.55]	0.03	4.16	0.75	[2.85, 5.68]
600	0.02	3.34	0.67	[2.26, 4.63]	0.03	4.05	0.56	[3.17, 5.16]	0.02	3.44	0.74	[2.19, 4.71]
700	0.02	2.69	0.55	[1.80, 3.82]	0.02	3.52	0.51	[2.59, 4.62]	0.02	2.81	0.53	[1.88, 3.73]
800	0.01	2.11	0.49	[1.31, 3.18]	0.02	3.05	0.42	[2.26, 3.91]	0.02	2.37	0.45	[1.65, 3.13]
900	0.01	1.70	0.41	[1.14, 2.67]	0.02	2.69	0.31	[2.12, 3.40]	0.01	2.04	0.41	[1.43, 3.01]

Table S24. Correlation matrix of the variable importance between the common variables of the three SML methods built using the subsets of spectra of the "Point" group.

	Gutter		Center		Margin	
	**PLS**	**RF**	**PLS**	**RF**	**PLS**	**RF**
**RF**	0.929		0.933	-	0.934	
**kNN**	0.931	0.999	0.927	0.999	0.941	0.999

Table S25. Summary of the relative contributions of squared bias and variance to MSE_{CV100} of PLS.

	PLS																			
	Publication date						Page						Point						All – No subsets	
	1851-1900		1901-1950		1951-2000		Front pages		Middle pages		Back pages		Gutter		Center		Margin			
**Number of spectra**	**Bias^2**	**Var**	**Bias^2**	**Var**	**Bias^2**	**Var**	**Bias^2**	**Var**	**Bias^2**	**Var**	**Bias^2**	**Var**	**Bias^2**	**Var**	**Bias^2**	**Var**	**Bias^2**	**Var**	**Bias^2**	**Var**
50	0.39	0.61	0.49	0.51	0.30	0.70	0.49	0.51	0.46	0.54	0.49	0.51	0.48	0.52	0.49	0.51	0.49	0.51	0.46	0.54
100	0.38	0.62	0.32	0.68	0.22	0.78	0.55	0.45	0.54	0.46	0.51	0.49	0.55	0.45	0.51	0.49	0.55	0.45	0.50	0.50
200	0.33	0.67	0.23	0.77	0.19	0.81	0.58	0.42	0.50	0.50	0.52	0.48	0.45	0.55	0.49	0.51	0.49	0.51	0.50	0.50
300	0.28	0.72	0.18	0.82	0.17	0.83	0.49	0.51	0.46	0.54	0.46	0.54	0.41	0.59	0.44	0.56	0.45	0.55	0.47	0.53
400	0.21	0.79	0.16	0.84	0.15	0.85	0.47	0.53	0.47	0.53	0.44	0.56	0.42	0.58	0.42	0.58	0.46	0.54	0.45	0.55
500	0.19	0.81	0.16	0.84	0.15	0.85	0.48	0.52	0.47	0.53	0.45	0.55	0.43	0.57	0.41	0.59	0.47	0.53	0.44	0.56
600	0.18	0.82	0.16	0.84	0.13	0.87	0.48	0.52	0.49	0.51	0.47	0.53	0.43	0.57	0.41	0.59	0.49	0.51	0.45	0.55
700	0.18	0.82	0.16	0.84	0.12	0.88	0.49	0.51	0.49	0.51	0.49	0.51	0.44	0.56	0.39	0.61	0.51	0.49	0.45	0.55
800	0.17	0.83	0.16	0.84	0.12	0.88	0.49	0.51	0.50	0.50	0.49	0.51	0.45	0.55	0.40	0.60	0.53	0.47	0.46	0.54
900	0.16	0.84	0.16	0.84	0.12	0.88	0.50	0.50	0.50	0.50	0.50	0.50	0.45	0.55	0.39	0.61	0.54	0.46	0.46	0.54

Table S26. Summary of the relative contributions of squared bias and variance to MSE_{CV100} of RF.

	RF																			
	Publication date						Page						Point						All – No subsets	
	1851-1900		1901-1950		1951-2000		Front pages		Middle pages		Back pages		Gutter		Center		Margin			
**Number of spectra**	**Bias^2**	**Var**	**Bias^2**	**Var**	**Bias^2**	**Var**	**Bias^2**	**Var**	**Bias^2**	**Var**	**Bias^2**	**Var**	**Bias^2**	**Var**	**Bias^2**	**Var**	**Bias^2**	**Var**	**Bias^2**	**Var**
50	0.75	0.25	0.83	0.17	0.77	0.23	0.80	0.20	0.80	0.20	0.80	0.20	0.79	0.21	0.78	0.22	0.80	0.20	0.80	0.20
100	0.72	0.28	0.79	0.21	0.72	0.28	0.79	0.21	0.79	0.21	0.79	0.21	0.78	0.22	0.78	0.22	0.79	0.21	0.78	0.22
200	0.69	0.31	0.75	0.25	0.67	0.33	0.78	0.22	0.78	0.22	0.79	0.21	0.75	0.25	0.75	0.25	0.77	0.23	0.77	0.23
300	0.65	0.35	0.72	0.28	0.65	0.35	0.77	0.23	0.76	0.24	0.77	0.23	0.73	0.27	0.73	0.27	0.75	0.25	0.75	0.25
400	0.62	0.38	0.69	0.31	0.63	0.37	0.75	0.25	0.75	0.25	0.77	0.23	0.71	0.29	0.72	0.28	0.74	0.26	0.74	0.26
500	0.60	0.40	0.66	0.34	0.61	0.39	0.75	0.25	0.74	0.26	0.77	0.23	0.70	0.30	0.71	0.29	0.73	0.27	0.73	0.27
600	0.58	0.42	0.64	0.36	0.60	0.40	0.73	0.27	0.74	0.26	0.76	0.24	0.67	0.33	0.70	0.30	0.71	0.29	0.71	0.29
700	0.57	0.43	0.63	0.37	0.59	0.41	0.73	0.27	0.73	0.27	0.76	0.24	0.65	0.35	0.69	0.31	0.70	0.30	0.70	0.30
800	0.56	0.44	0.61	0.39	0.58	0.42	0.72	0.28	0.72	0.28	0.76	0.24	0.64	0.36	0.68	0.32	0.68	0.32	0.70	0.30
900	0.55	0.45	0.60	0.40	0.56	0.44	0.71	0.29	0.72	0.28	0.76	0.24	0.62	0.38	0.67	0.33	0.67	0.33	0.68	0.32

Table S27. Summary of the relative contributions of squared bias and variance to MSE_{CV100} of kNN.

	kNN																			
	Publication date						Page						Point						All – No subsets	
	1851-1900		1901-1950		1951-2000		Front pages		Middle pages		Back pages		Gutter		Center		Margin			
**Number of spectra**	**Bias²**	**Var**	**Bias²**	**Var**	**Bias²**	**Var**	**Bias²**	**Var**	**Bias²**	**Var**	**Bias²**	**Var**	**Bias²**	**Var**	**Bias²**	**Var**	**Bias²**	**Var**	**Bias²**	**Var**
50	0.36	0.64	0.48	0.52	0.41	0.59	0.48	0.52	0.49	0.51	0.49	0.51	0.48	0.52	0.44	0.56	0.46	0.54	0.48	0.52
100	0.26	0.74	0.32	0.68	0.28	0.72	0.44	0.56	0.44	0.56	0.42	0.58	0.39	0.61	0.39	0.61	0.37	0.63	0.39	0.61
200	0.17	0.83	0.17	0.83	0.16	0.84	0.36	0.64	0.35	0.65	0.36	0.64	0.26	0.74	0.28	0.72	0.24	0.76	0.32	0.68
300	0.13	0.87	0.11	0.89	0.12	0.88	0.29	0.71	0.28	0.72	0.29	0.71	0.19	0.81	0.20	0.80	0.17	0.83	0.26	0.74
400	0.11	0.89	0.08	0.92	0.10	0.90	0.23	0.77	0.23	0.77	0.24	0.76	0.13	0.87	0.14	0.86	0.12	0.88	0.20	0.80
500	0.10	0.90	0.06	0.94	0.09	0.91	0.19	0.81	0.19	0.81	0.21	0.79	0.10	0.90	0.14	0.86	0.12	0.88	0.17	0.83
600	0.09	0.91	0.05	0.95	0.08	0.92	0.16	0.84	0.16	0.84	0.17	0.83	0.10	0.90	0.13	0.87	0.11	0.89	0.15	0.85
700	0.09	0.91	0.04	0.96	0.07	0.93	0.14	0.86	0.13	0.87	0.15	0.85	0.09	0.91	0.11	0.89	0.11	0.89	0.12	0.88
800	0.08	0.92	0.04	0.96	0.07	0.93	0.13	0.87	0.12	0.88	0.14	0.86	0.08	0.92	0.10	0.90	0.10	0.90	0.11	0.89
900	0.08	0.92	0.03	0.97	0.06	0.94	0.13	0.87	0.11	0.89	0.14	0.86	0.09	0.91	0.10	0.90	0.11	0.89	0.09	0.91

S5 Supplementary References

[1] Trafela, T.; Strlič, M.; Kolar, J.; Lichtblau, D. A.; Anders, M.; Mencigar, D. P.; Pihlar, B. Nondestructive Analysis and Dating of Historical Paper Based on IR Spectroscopy and Chemometric Data Evaluation. *Anal. Chem.* **2007**, *79* (16), 6319–6323. DOI: 10.1021/ac070392t.

[2] Brown, N.; Lichtblau, D.; Fearn, T.; Strlič, M. Characterisation of 19th and 20th Century Chinese Paper. *Heritage Sci.* **2017**, *5* (47), 1–14. DOI: 10.1186/s40494-017-0158-x.

[3] Silva, C. S.; Pimentel, M. F.; Amigo, J. M.; García-Ruiz, C.; Ortega-Ojeda, F. Chemometric Approaches for Document Dating: Handling Paper Variability. *Anal. Chim. Acta* **2018**, *1031*, 28–37. DOI: 10.1016/j.aca.2018.06.031.

[4] NUK, "National and University Library of Slovenia. Website." can be found under https://www.nuk.uni-lj.si/eng/, **2022** (accessed 17 February 2023).

[5] Pope, J. M. Near-Infrared Spectroscopy of Wood Products. In *Surface Analysis of Paper*; Conners, T. E., Banerjee, S., Eds.; CRC Press, 1995; pp 142–151.

[6] Wickham, H.; Averick, M.; Bryan, J.; Chang, W.; McGowan, L. D.; François, R.; Grolemund, G.; Hayes, A.; Henry, L.; Hester, J.; Kuhn, M.; Pedersen, T. L.; Miller, E.; Bache, S. M.; Müller, K.; Ooms, J.; Robinson, D.; Seidel, D. P.; Spinu, V.; Takahashi, K.; Vaughan, D.; Wilke, C.; Woo, K.; Yutani, H. Welcome to the Tidyverse. *Journal of Open Source Software* **2019**, *4*, 1686. DOI: 10.21105/joss.01686.

[7] Stevens, A.; Ramirez-Lopez, L.; Hans, G. An introduction to the prospectr package. *R Package Vignette* **2022**.

[8] Scrucca, L. GA: A Package for Genetic Algorithms in R. *Journal of Statistical Software* **2013**, *53*, 1–37. DOI: 10.18637/jss.v053.i04.

[9] Kursa, M. B.; Rudnicki, W. R. Feature Selection with the Boruta Package. *Journal of Statistical Software* **2010**, *36*, 1–13. DOI: 10.18637/jss.v036.i11

[10] Liland, K. H.; Mevik, B.-H.; Wehrens, R.; Hiemstra, P. pls: Partial Least Squares and Principal Component Regression. *R package* **2022**.

[11] Wright, M. N.; Ziegler, A. ranger: A Fast Implementation of Random Forests for High Dimensional Data in C++ and R. *Journal of Statistical Software* **2017**, *77*, 1–17. DOI: 10.18637/jss.v077.i01

[12] Bischl, B.; Lang, M.; Kotthoff, L.; Schiffner, J.; Richter, J.; Studerus, E.; Casalicchio, G.; Jones, Z. M. mlr: Machine Learning in R. *Journal of Machine Learning Research* **2016**, *17*, 1–5.

[13] Rinnan, Å.; Berg, F. van den; Engelsen, S. B. Review of the Most Common Pre-Processing Techniques for near-Infrared Spectra. *TrAC, Trends Anal. Chem.* **2009**, *28* (10), 1201–1222. DOI: 10.1016/j.trac.2009.07.007.

[14] Savitzky, Abraham.; Golay, M. J. E. Smoothing and Differentiation of Data by Simplified Least Squares Procedures. *Anal. Chem.* **1964**, *36* (8), 1627–1639. DOI: 10.1021/ac60214a047.

[15] Svetnik, V.; Liaw, A.; Tong, C.; Culberson, J. C.; Sheridan, R. P.; Feuston, B. P. Random Forest: A Classification and Regression Tool for Compound Classification and QSAR Modeling. *J. Chem. Inf. Comput. Sci.* **2003**, *43*, 1947–1958. DOI: 10.1021/ci034160g
